# Insights into the ecology of *Schizosaccharomyces* species in natural and artificial habitats

**DOI:** 10.1007/s10482-022-01720-0

**Published:** 2022-03-31

**Authors:** Michael Brysch-Herzberg, Guo-Song Jia, Martin Seidel, Imen Assali, Li-Lin Du

**Affiliations:** 1 Laboratory for Wine Microbiology, Department International Business, Heilbronn University, Max-Planck-Str. 39, 74081 Heilbronn, Germany; 2 National Institute of Biological Sciences, Beijing, 102206 China; 3 Tsinghua Institute of Multidisciplinary Biomedical Research, Tsinghua University, Beijing, 102206 China; 4 Department of Bioengineering, National Engineering School of Sfax, University of Sfax, Soukra, km 4, 3038 Sfax, Tunisia

**Keywords:** *Schizosaccharomyces*, Isolation method, Enrichment, Ecology

## Abstract

The fission yeast genus *Schizosaccharomyces* contains important model organisms for biological research. In particular, *S. pombe* is a widely used model eukaryote. So far little is known about the natural and artificial habitats of species in this genus. Finding out where *S. pombe* and other fission yeast species occur and how they live in their habitats can promote better understanding of their biology. Here we investigate in which substrates *S. pombe*, *S. octosporus*, *S. osmophilus* and *S. japonicus* are present. To this end about 2100 samples consisting of soil, tree sap fluxes, fresh fruit, dried fruit, honey, cacao beans, molasses and other substrates were analyzed. Effective isolation methods that allow efficient isolation of the above mentioned species were developed. Based on the frequency of isolating different fission yeast species in various substrates and on extensive literature survey, conclusions are drawn on their ecology. The results suggest that the primary habitat of *S. pombe* and *S. octosporus* is honeybee honey. Both species were also frequently detected on certain dried fruit like raisins, mango or pineapple to which they could be brought by the honey bees during ripening or during drying. While *S. pombe* was regularly isolated from grape mash and from fermented raw cacao beans *S. octosporus* was never isolated from fresh fruit. The main habitat of *S. osmophilus* seems to be solitary bee beebread. It was rarely isolated from raisins. *S. japonicus* was mainly found in forest substrates although it occurs on fruit and in fruit fermentations, too.

**Supplementary Information:**

The online version contains supplementary material available at 10.1007/s10482-022-01720-0.

## Introduction

The genus *Schizosaccharomyces* currently consists of 5 species: *S. pombe* (Lindner 1893), *S. octosporus* (Beijerinck 1894), *S. japonicus* (Yukawa and Maki 1931), *S. cryophilus* (Helston et al. 2010) and *S. osmophilus* (Brysch-Herzberg et al. 2019). This genus belongs to the subphylum *Taphrinomycotina*, which is basal to the subphyla *Saccharomycotina* and *Pezizomycotina* of the phylum *Ascomycota* (Shen et al. 2020; Sipiczki 1995).

The most prominent species in this genus, *S. pombe*, was first used for genetic studies by Urs Leupold in the 1940s (Leupold 1950), and has since become one of a handful of most important eukaryotic model species for molecular and cellular biology (Fantes and Hoffman 2016; Hayles and Nurse 2018; Hoffman et al. 2015). Like the other widely used model yeast, the budding yeast *Saccharomyces cerevisiae*, *S. pombe* has many advantages as an experimental organism, including ease of culturing, powerful genetic tools, and a compact genome. Due to the large evolutionary distance between *S. pombe* and *S. cerevisiae* (they are estimated to have diverged more than 500 million years ago (Shen et al. 2020)), these two model yeasts are distinct from each other in many aspects of cellular components and organization. For example, like animals, *S. pombe* has large centromeres, high percentage of genes containing introns, histone H3K9 methylation-based heterochromatin, and the RNAi machinery, whereas *S. cerevisiae* has point centromeres, few introns, and neither H3K9 methylation nor RNAi. Moreover, many vertebrate genes have orthologues in *S. pombe* but not *S. cerevisiae* (Aravind et al. 2000; Hoffman et al. 2015; Sipiczki 2000). Thus, only *S. pombe* but not *S. cerevisiae* is suitable for studying the functions of these genes. In addition to *S. pombe*, another fission yeast species, *S. japonicus*, has been developed into a model organism in the last decade for studying cell biology (Klar 2013; Russell et al. 2017).

Despite the deep knowledge in cellular biology and genetics of *S. pombe*, “remarkably little is known about the evolutionary history or ecology of this model organism.” (Jeffares et al. 2015). This is due to the major difficulties associated with the targeted isolation of *S. pombe* from nature. Although methods for the isolation of *S. pombe* from special substrates like honey and honeycombs (Benito et al. 2013) and grapes (Balloni and Pelosi 1977; Florenzano et al. 1977) have been developed, no large scale isolation trial has been performed yet.

As a consequence of the lack of systematic global isolation trials, virtually all *S. pombe* strains that have been investigated so far were isolated from substrates related to human activities (Jeffares 2018). Population genomic studies on currently available *S. pombe* strains have revealed a pattern of recent and possibly human-caused admixture between two distinct ancestral lineages (Jeffares et al. 2015; Tao et al. 2019; Tusso et al. 2019). But such a pattern may result from biased sampling and is unlikely to reflect the situations of truly wild natural populations.

Gaining additional knowledge on the occurrence and ecology of fission yeast species may help us understand the evolutionary selection forces that have shaped the species as we know them today. The targeted isolation of new strains from local populations as well as on a global scale could lead to a deeper understanding of the evolutionary history of *Schizosaccharomyces* species.

None of the *Schizosaccharomyces* species is ubiquitously distributed. The scarce isolation of these species suggests a highly specialized life history. They seem to rarely dominate the microbial communities in which they are found.

In the current study it was first necessary to develop adequate methods for the isolation of the different *Schizosaccharomyces* species from the various substrates which were investigated.

Most of the substrates from which the species have been isolated in the past are not distributed over all continents and all climate zones. For example, *S. pombe* has been isolated sporadically from wine grape related substrates. Vine (*Vitis vinifera*) grows in subtropical and warm parts of temperate regions but not in tropical or cold regions. Similarly, *S. pombe* was shown to be a typical member of the yeast communities in cacao fermentations and the cacao tree (*Theobroma cacao*) is cultivated in the humid tropics and does not grow in other climate zones. Therefore, identification of substrates that would allow the targeted isolation of *Schizosaccharomyces* species is a necessary step towards worldwide sampling of *S. pombe* and other fission yeast species.

The aim of the current study was to increase the understanding of the life history of the different fission yeast species, both in natural and artificial habitats. This is achieved by the development of effective isolation methods, large-scale isolation attempts on many different kinds of substrates and an extensive survey of the available literature.

## Materials and methods

In the current study a total of more than 2132 samples were investigated. Different enrichment media were used for most of them but it was virtually impossible to use every medium for every sample. Therefore, initially, for each substrate investigated, a set of enrichment media which seemed promising in connection with the substrate and the target species was tested. If one or more of the media turned out to be effective for the isolation of one or more *Schizosaccharomyces* species, the medium/the media was/were routinely used for a larger number of samples of the same substrate.

### Preparation of media

The composition of all enrichment media routinely used in the current investigation is given in Table 1. For preparation of medium CY, peptone and yeast extract were autoclaved separately from the other components of the medium in order to prevent intensive browning. Ethanol, acetic acid and sterile filter-sterilized cycloheximide solution were added under aseptic conditions after autoclaving.

**Table 1:** Media used in the current investigation

Medium^a^	Glucose	Fructose	Cyclo-heximide	Ethanol	Acetic acid	NaCl	Chloram-phenicol	Pepton	Yeast extract
FG^b^	5% (w/w)	55% (w/w)	–	–	–	–	–	0.5 (w/v)	0.5 (w/v)
Na^b^	2% (w/v)	–	–	–	–	16% (w/v)	0.01 (w/v)	0.5 (w/v)	0.5 (w/v)
O	2%	–	–	8% (w/v)	–	–	0.01 (w/v)	0.5 (w/v)	0.5 (w/v)
CY	2%	–	0.001 (w/v)	6% (w/v)	0.8% (w/v)		0.01 (w/v)	0.5 (w/v)	0.5 (w/v)
MC	2%	–	0.001 (w/v)	6% (w/v)			0.01 (w/v)	0.5 (w/v)	0.5 (w/v)
YPD^b^	2%	–					0.01 (w/v)	0.5 (w/v)	0.5 (w/v)

^a^ Basis for all media is YPD medium (0.5% yeast extract, 0.5% pepton, 2% glucose) ^b^ Oil was layered on top of the medium to curb mold growth

Medium FG was prepared by dissolving glucose and fructose in water. This solution was autoclaved separately from the solution of peptone/yeast extract. After autoclaving at 115 °C for 15 min both solutions were mixed under aseptic conditions. Fructose was used as predominating sugar because of its better solubility in water. The authors observed that media containing 60% glucose crystalize easily when they get in contact with sample material whereas the medium described here does not. Fructose can be used to substitute glucose in a medium without substantially altering the osmotic pressure of the medium (Ebrahimi and Sadeghi 2016; Tokuoka et al. 1985).

Medium O was prepared by autoclaving all constituents except ethanol for 15 min at 121 °C. Ethanol was added after autoclaving under aseptic conditions. For preparation of media YPD and Na all constituents were autoclaved for 15 min at 121 °C. For the preparation of medium MC filter-sterilized cycloheximide solution was added to YPD medium.

#### Sampling and processing of grape mash

Several media were tested for their utility to enrich *S. pombe* from fresh fruit, fruit mash, fruit musts, rotting fruit and soil. The different media were based on the use of different growth inhibitors such as SO_2_ (600 mg/l K_2_S_2_O_5_ equivalent to approximately 300 mg/l SO_2_), the combination of cycloheximide (0.001%) and benzoic acid (0.035%), the combination of acetic acid (0.8%) and ethanol (6%) and the combination of acetic acid (0.8%), ethanol (6%) and cycloheximide (0.001%).

Grape mash samples were taken with and transported in 200 ml sterile plastic screw cap beakers. Grape mash was taken from the production line directly after destemming. 100 ml of fruit mash were filled in 250 ml screw cap glass bottles. 100 ml of two times concentrated CY medium were added and thoroughly mixed with the fruit mash by shaking. Incubation took place at 28 °C for at least 2 months before a sample was recorded as negative for *S. pombe*.

257 and 156 grape mash samples were investigated in 2017 and in 2020, respectively. All samples were taken in vine growers cooperatives (n = 11) or from independent wineries (n = 5) in the vine growing region Württemberg, Germany. Each sample was part of a new grape delivery by the vine growers.

#### Sampling and processing of fruit, fruit must and rotting fruit

Field samples were taken aseptically, stored in sterile polyethylene zipper bags and transported in a cool box to the lab and processed at the same day. Fruit mash was produced from the collected fruit by thoroughly squeezing the fruit within the polyethylene bags in which they were collected if the consistence of the fruit allowed squeezing. Hard fruit such as healthy apples or pears were pureed in a sterile mixer. In the case of medium CY 100 ml of two times concentrated medium was mixed with 100 ml of the sample. In the case of all other enrichment media 10 ml of sample were mixed with 190 ml of medium. All samples were incubated at 28 °C. Incubation took place at 28 °C. In case no fermentation was observed samples were incubated for at least 2 months.

In three different fruit juice production facilities 6 apple juice samples taken directly after pressing of the apples during the production of apple juice were examined.

From mounds of rotting apples samples were taken in a depth of about 10–25 cm where high numbers of insect larvae were present and the fermenting apples already lost their form and cohesion. 35 samples from 7 different apple growers in Germany were taken. 6 apple growers were located in Württemberg and one in Rheinhessen, Germany. Six rotting apple samples were collected in Changping District, Beijing, China. Four rotting apple samples were collected in Laiyang City, Shandong Province, China. Table 2 provides an overview of all fresh fruit, fruit must and rotting fruit samples.

**Table 2:** Substrates investigated and species detected by means of different enrichment media

		Total				YPD				CY				O				FG				Na				MC			
		Sp	Sc	Ss	Sj	Sp	Sc	Ss	Sj	Sp	Sc	Ss	Sj	Sp	Sc	Ss	Sj	Sp	Sc	Ss	Sj	Sp	Sc	Ss	Sj	Sp	Sc	Ss	Sp
Grape mash	n	413				−				413				−				−				−				−			
	+	69	0	0	0	−	−	−	−	69	0	0	0	−	−	−	−	−	−	−	−	−	−	−	−	−	−	−	−
	%	16.7	0	0	0	−	−	−	−	16.7	0	0	0	−	−	−	−	−	−	−	−	−	−	−	−	−	−	−	−
Grapes collected in the vineyard	n	115				−				30				24				61				−				−			
	+	0	0	0	0	−	−	−	−	0	0	0	0	0	0	0	0	0	0	0	0	−	−	−	−	−	−	−	−
	%	0	0	0	0	−	−	−	−	0	0	0	0	0	0	0	0	0	0	0	0	−	−	−	−	−	−	−	−
Composted apples	n	45				−				45				11				11				11				−			
	+	19	0	0	0	−	−	−	−	19	0	0	0	0	0	0	0	0	0	0	0	0	0	0	0	−	−	−	−
	%	42.2	0	0	0	−	−	−	−	42.2	0	0	0	0	0	0	0	0	0	0	0	0	0	0	0	−	−	−	−
Fresh or half rotten apples under apple trees	n	35				−				35				−				−				−				−			
	+	S	0	0	0	−	−	−	−	S	0	0	0	−	−	−	−	−	−	−	−	−	−	−	−	−	−	−	−
	%	−	0	0	0	−	−	−	−	−	0	0	0	−	−	−	−	−	−	−	−	−	−	−	−	−	−	−	−
Apple must	n	6				−				6				−				−				−				−			
	+	S	0	0	0	−	−	−	−	S	0	0	0	−	−	−	−	−	−	−	−	−	−	−	−	−	−	−	−
	%	−	0	0	0	−	−	−	−	−	0	0	0	−	−	−	−	−	−	−	−	−	−	−	−	−	−	−	−
Various fresh fruit (other than grapes and apple)	n	134				−				45				80^a^				22				9				−			
	+	0	0	0	S	−	−	−	−	0	0	0	0	0	0	0	S	0	0	0	0	0	0	0	0	−	−	−	−
	%	0	0	0	0	−	−	−	−	0	0	0	0	0	0	0	0	0	0	0	0	0	0	0	0	−	−	−	−
Vineyard soil	n	38				−				20				18				−				−				−			
	+	0	0	0	0	−	−	−	−	0	0	0	0	0	0	0	0	−	−	−	−	−	−	−	−	−	−	−	−
	%	0	0	0	0	−	−	−	−	0	0	0	0	0	0	0	0	−	−	−	−	−	−	−	−	−	−	−	−
Soil under fruit bearing tree	n	32				−				32				28				−				−				−			
	+	S	0	0	0	−	−	−	−	S	0	0	0	0	0	0	0	−	−	−	−	−	−	−	−	−	−	−	−
	%	−	0	0	0	−	−	−	−	−	0	0	0	0	0	0	0	−	−	−	−	−	−	−	−	−	−	−	−
Bark and flux of fruit bearing tree outside the forest	n	51				−				−				51^a^				−				−				−			
	+	0	0	0	2	−	−	−	−	−	−	−	−	0	0	0	2	−	−	−	−	−	−	−	−	−	−	−	−
	%	0	0	0	3.9	−	−	−	−	−	−	−	−	0	0	0	3.9	−	−	−	−	−	−	−	−	−	−	−	−
Dried fruit	n	430				−				20				303				430				−				−			
	+	35	88	3		−	−	−	−	0	0	0	0	27	31	0	0	9	73	3	0	−	−	−	−	−	−	−	−
	%	8.5	21.3	0.8		−	−	−	−	0	0	0	0	9.5	10.9	0	0	2.1	17.0	0.9	0	−	−	−	−	−	−	−	−
Honey	n	386				386				54				386				386				192				−			
	+	49	61	S	0	27	37	0	0	0	0	0	0	31	28	0	0	2	30	S	0	0	7	0	0	−	−	−	−
	%	12.7	15.8	−	0	7.0	9.6	0	0	0	0	0	0	8.0	7.3	0	0	0.5	7.8	−	0	0	3.6	0	0	−	−	−	−
Bee hive material	n	37				−				29				37				37				37				−			
	+	S	0	0	0	0	0	0	0	0	0	0	0	S	0	0	0	0	0	0	0	0	0	0	0	−	−	−	−
	%	−	0	0	0	0	0	0	0	0	0	0	0	−	0	0	0	0	0	0	0	0	0	0	0	−	−	−	−
Solitary bee beebread	n	123				−				21				30				123				42				−			
	+	0	S	14	0	0	0	0	0	0	0	0	0	0	0	0	0	0	S	14	0	0	0	0	0	−	−	−	−
	%	0	−	11.4	0	0	0	0	0	0	0	0	0	0	0	0	0	0	−	11.4	0	0	0	0	0	−	−	−	−
Forest materials (Bark, slime flux, soil)	n	219				−				41				252^b^				41				41				−			
	+	0	0	0	11	−	−	−	−	0	0	0	0	0	0	0	11	0	0	0	0	0	0	0	0	−	−	−	−
	%	0	0	0	5.02	−	−	−	−	0	0	0	0	0	0	0	4.4	0	0	0	0	0	0	0	0	−	−	−	−
Cacao beans (whole beans, nibbs and powder)	n	75				75^a^				−				−				−				−				75			
	+	50	0	0	0	32	−	−	−	−	−	−	−	−	−	−	−	−	−	−	−	−	−	−	−	46	0	0	0
	%	66.7	0	0	0	42.7	−	−	−	−	−	−	−	−	−	−	−	−	−	−	−	−	−	−	−	61.3	0	0	0

The frequency of isolation of each species is given per substrate and per medium absolutely (+) and as percent (%)

For each medium and substrate, the number of samples (n) investigated is given

*Samples from 9 sites, 2 in China and 7 in Germany (*S. pombe* detected on all German and 1 of 2 Chinese sites)

^a^Incubated at 37 °C; *S* single isolation, no percent value calculated; − = not investigated

^b^Samples investigated byBrysch-Herzberg and Seidel (2017)

#### Sampling and processing of honey

386 commercial honeys from 43 countries in different regions of the world were purchased. Details about the samples investigated are given in Supplementary Table 3.

The honey and honey comb containers were opened under aseptic conditions. 5 ml sample volumes were mixed thoroughly with 45 ml of medium in 50 ml plastic centrifuge screw cap tubes. Initially, all aliquots of all samples were incubated in media YPD, O, FG, Na and CY. After incubation of 54 honeys no *S. pombe* was isolated by means of medium CY. Thus use of medium CY for honey samples was discontinued. After incubation of 192 honeys it became apparent that use of medium Na is inferior to the use of other media for the enrichment of *S. octosporus*. It was not used any more. The remaining 194 samples were all incubated in media YPD, FG and O, only. The number of samples investigated per country are given in Supplementary Table 1.

#### Sampling and processing of dried fruit, cacao products and coffee beans

Dried fruit of many kinds, raw, unroasted cacao beans, cacao nibs, cacao powder and unroasted coffee beans from various countries were bought. Packages were opened under aseptic conditions. About 50 ml of sample material was poured in 200 ml sterile plastic screw cap beakers. The beakers were filled up to a volume of 200 ml with enrichment medium. Dried fruit were incubated in media FG and O while cacao products and coffee beans were incubated in media YPD and MC. Incubation took place at 28 °C. The only exception were cacao products and coffee beans in medium YPD. These were incubated at 37 °C. Supplementary Table 2 provides an overview of the kind of fruit investigated and their origins. In Supplementary Table 3 cacao product samples investigated and their origins are given.

#### Sampling and processing of soil samples

Soil samples were taken aseptically from the upper 5 cm of soil including litter if present. Samples were stored and transported in sterile polyethylene zipper bags. Under aseptic conditions in the lab approximately 75 ml of sample material were placed in a 250 ml screw cap glass bottles. In case that CY medium was used the volume was filled up to 100 ml with 0.8% malic acid. Malic acid was applied in order to eliminate carbonate from the soil and thus keep the pH low. A low pH is a prerequisite for the antimicrobial activity of acetic acid. After adding 100 ml of two times concentrated CY medium the samples were incubated as described above. In the case of all other enrichment media after filling 75 ml of sample material in the glass bottle the volume was filled up to 150 ml with the respective enrichment medium. After shaking the samples were incubated at 28 °C. Soil samples were taken in the forest, in vineyards and under fruit-bearing trees. Table 2 provides an overview of the soil samples investigated.

#### Sampling and processing of bee hive material

Samples were taken from beehives of two beekeepers in Württemberg, Germany (29 samples) and one beekeeper in Canterbury, Great Britain (8 samples). The German samples came from 12 hives at 4 different locations. The British samples all came from 4 hives at the same location. Per hive one sample of honeycombs and one sample of combs with bee bread were taken. Additionally, old comb material intended for candle wax production was investigated. For enrichment small pieces of comb material of about 2 cm × 4 cm were cut with a sterile knife and placed in 50 ml screw cap plastic centrifuge tubes. If necessary, the combs were squeezed open with a sterile spatula. After filling a tube with enrichment medium it was shaken until all materials in the combs were washed out of the combs. Aliquots of each German sample were incubated with the media CY, FG, O and Na. After none of the German samples had started fermentation in medium CY the British samples were incubated with media FG, O and Na. Incubation was continued for a minimum time of 2 months at 25 °C if no fermentation occurred.

#### Sampling and processing of sugar cane molasses

A total of 17 samples of sugar cane molasses coming from 1 Dutch and 5 German sugar production plants were kindly provided by a German sugar production company. The samples were partly taken from storage tanks and the production process. All samples were treated as described for honey above. Samples were incubated in media YPD, O and FG.

#### Sampling and processing of forest samples

Forest soil samples were taken as described above. Samples of bark were taken from black spots, lesions and slime fluxes as described before (Brysch-Herzberg and Seidel 2017). Aliquots of all samples were incubated in media CY, O, FG and Na. Because of the omnipresence of molds in the forest materials media FG and Na were covered with oil during incubation in order to slow down the growth of air dependent organisms like molds. Medium O was not applied to all forest samples since the authors have investigated hundreds of samples by use of this medium before (Brysch-Herzberg and Seidel 2017).

#### Sampling and processing of solitary bee beebread

Solitary bees were offered nesting material consisting of bamboo sticks, carton tubes and natural straws. The lumen ranged between 1 and 12 mm. Bundles of the tubes were placed at a dry place in direct sunshine in an environment rich of different flowering plants. In case a bee capped the end of a tube the tube was taken to the lab, cut open under aseptic conditions and the beebread material was investigated. The bee bread from different chambers of a tube was treated as one sample. Aliquots of each sample were placed in 50 ml screw cap plastic centrifuge tubes and filled with enrichment medium. The beebread was suspended in the medium by shaking. Aliquots were incubated in media CY, FG, Na, O and YPD medium. Incubation was done at least for two months at 25 °C or until fermentation took place. Table 2 provides information about aliquots of how many samples were treated with which of the above mentioned media.

#### Sampling and processing of cultures for tea fermentation

So called “Kombucha” starter cultures sold as layers of the microbial consortium typical for tea fermentations were purchased from internet shops. After shaking 100 µl of the liquid were added to medium MC in 50 ml screw cap plastic centrifuge tubes and incubated at 25 °C for at least 2 months.

### Isolation

In case gas formation started during incubation of the samples in the enrichment media the material was examined microscopically. In case fission yeast cells were observed under the microscope isolation was done by streaking 3 µl of the medium on YPD agar. In case honey or dried fruit were incubated in the enrichment medium it was suspected that some strains could be obligate osmophiles. Therefore, in the case of honey and dried fruit streaking was done on YPD agar supplemented with 5% glucose and 25% fructose. In subsequent inoculation steps it was tested if the strains could grow on standard YPD agar as well. If after streaking of the enrichment medium fission yeast colonies were distinguishable by macroscopic and/or microscopic characteristics more than one colony type was further cultured and subsequently purified by streaking. All strains were identified by D1/D2 sequence analysis as given below.

#### Identification of yeast isolates

All fission yeast strains isolated were identified by analysis of the D1/D2 domain of the large subunit rDNA gene (D1/D2 domain). During isolation method development yeast “bycatch” was identified randomly by D1/D2 sequencing as well. The methods for DNA isolation, amplification of the D1/D2 domain have been described earlier (Brysch-Herzberg and Seidel 2015). With the new sequences a blast search was performed against Genbank. Alignments with the closest related type strain sequences revealed the identity of the newly isolated strains.

## Results

### Development of an enrichment medium for *S. pombe* from fruit and soil materials

#### SO_2_

In all sulfited samples in which fermentations started, *Saccharomycodes ludwigii* was the predominant species. It could easily be identified under the microscope by its cell shape and bipolar mode of budding. For some of the strains arbitrarily selected, the micromorphological identification was verified by sequence analysis of the D1/D2 domain. Besides, a budding yeast growing in flocs, which was identified at random by D1/D2 sequence analysis as *Zygosaccharomyces bailii* was observed frequently too. Fission yeast cells were not observed at all in sulfite-containing media.

#### Benzoic acid and cycloheximide

In samples of half rotten apples and soils from apple plantings incubated in the medium developed by Benito et al. (2013) for the isolation of *S. pombe* from honey, unidentified arthroconidia forming fungi, film forming yeasts and some molds grew in all samples. No fission yeast cells were observed.

#### Ethanol and acetic acid

The microscopic examination of enrichment cultures that exhibited gas formation as a sign of fermentation revealed that the predominant species were *Sa. ludwigii* and *Z. bailii*. However, in 5 of 42 enrichment cultures, fission yeast cells were observed and isolated. D1/D2 domain sequence analysis showed that all 5 cultures were strains of *S. pombe*.

#### Ethanol, acetic acid and cycloheximide

Pretests with the CY-medium including grape mash samples, other fruit samples and soil samples as substrate (twenty each) showed that the medium was highly specific for *S. pombe*. In the first 2–3 days of incubation, sometimes faint gas formation was observed that ceased soon. False positive samples showing fermentation but not harboring *S. pombe* hardly occurred at all (3 out of 791). CY medium was used for all grape mash, fresh fruit, rotten fruit and soil samples investigated in the current study.

### Isolation of *Schizosaccharomyces* species from fresh fruit, fruit mash, fruit must, rotting fruit and related soils

The results of the isolation trials are given in Table 2. In 2017, 34 *S. pombe* strains were isolated from 257 grape mash samples (~ 13%) while in 2020, 35 of 156 (~ 21%) samples contained *S. pombe*. All these samples were incubated in medium CY.

In Germany, out of 35 samples taken from mounds of rotting apples, 18 contained *S. pombe*. *S. pombe* was detected on all 7 sites belonging to different apple growers. In China, *S. pombe* was isolated from 4 out of 6 rotting apple samples collected at one location (Changping District, Beijing) but not from rotting apple samples collected at another location (Laiyang City, Shandong Province).

### Forest samples

31 samples of bark and slime flux materials collected at different locations from different tree species including *Quercus robur, Fagus sylvatica, Prunus avium, Carpinus betulus, Acer campestre* were investigated. Additionally, 10 samples of forest soil collected under *Quercus robur, Abies alba* and *Picea abies* were examined. Aliquots of all 41 samples were incubated in media CY, O, FG and NA. None of the samples yielded *Schizosaccharomyces* species.

### Unpublished results of earlier investigations

Brysch-Herzberg and Seidel (2017) incubated a total of 423 samples in medium O. Only the data from sampling areas Ruwer-Valley and Oberolmer Forest (see Brysch-Herzberg and Seidel 2017) are considered here in connection with *S. japonicus* because from all other samples the authors did not isolate dimorphic fungi. In 5 out of 64 (7.8%) forest soil samples *S. japonicus* was detected. From the gum like fluxes of *Prunus avium* (cherry tree), 55 samples were investigated, of which 5 (9.1%) contained *S. japonicus*. 2 of 21 (9.5%) fruit samples and 6 out of 43 (14.0%) bark and sap flux samples contained *S. japonicus*. In addition to the samples related to the above mentioned study, the authors investigated 49 samples of forest materials collected at different sites in Provence, France. 4 of these samples yielded *S. japonicus*.

### Isolation of *Schizosaccharomyces* species from dried fruit

A total of 429 dried fruit samples were investigated. All samples were enriched using medium FG. Of those, 303 samples were also enriched with medium O. 20 samples were incubated in medium CY. With medium CY no fermentation occurred. Thus, the medium was not used any more for dried fruit.

**Fig. 1:**
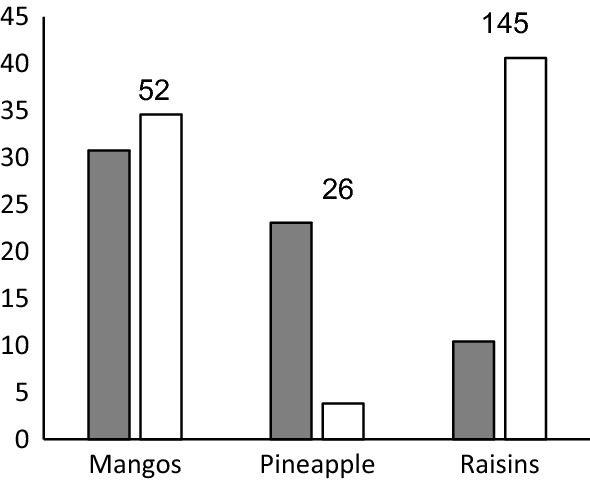
Frequency [%] of *S. pombe* and *S. octosporus* in samples of different kind of dried fruit. The total number of samples of each kind of fruit is given above the columns. grey: *S. pombe*, white: *S. octosporus*

While *S. octosporus* was more frequently found than *S. pombe* in raisin samples, pineapple samples yielded more often *S. pombe* than *S. octosporus*. From mango samples both species were isolated with about the same frequency (Fig. 1). From all other kinds of fruit, the number of isolations was only 1 or 2 which does not allow a sensible comparison of the frequency of both yeast species for these fruit. *S. osmophilus* was isolated two times from raisin samples and two times from dried crabapple samples (*Malus* × *robusta*). The successful isolation of *S. osmophilus* from one Chinese crabapple sample was performed in Germany in 2019. subsequently 17 other crabapple samples were investigated in China in 2020, and only one sample yielded *S. osmophilus*.

**Fig. 2:**
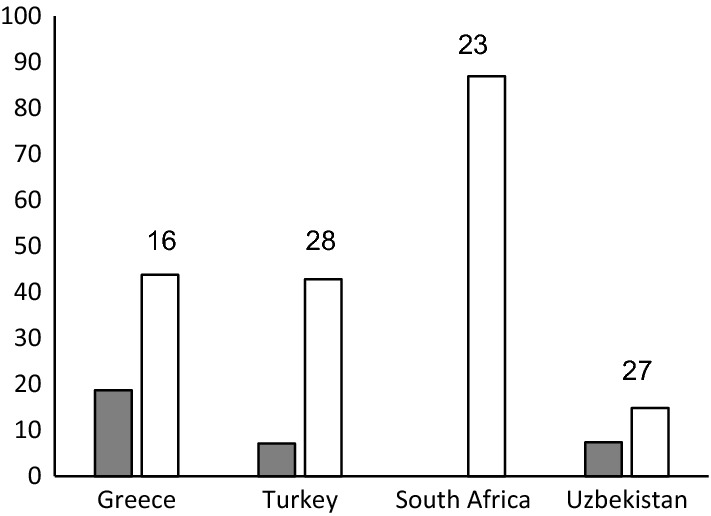
Frequency [%] of *S. pombe* (grey) and *S. octosporus* (white) in raisin samples from different countries. The number of samples per country is given above the columns

The success rate for the isolation of *S. octosporus* and *S. pombe* from the same kind of fruit differed between countries. While from South African raisin samples (n = 23) *S. pombe* was not isolated at all, it was isolated from 18.8% of Greek raisin samples (n = 16). *S. octosporus* was detected in 87.0% of the South African samples and in 14.8% of the Uzbek samples (n = 27). The frequency of the two species in raisin samples from additional countries is given in Fig. 2. *Schizosaccharomyces osmophilus* was isolated once from raisins from Iran and once from raisins in Uzbekistan. Raisins were the only fruit from which enough samples were examined to do a comparison by country.

### Honey

A total of 386 different honeys from 43 countries were investigated. *S. octosporus* was isolated from 61 (15.8%), *S. pombe* from 49 (12.7%), and *S. osmophilus* from 1 (0.26%) honey sample. 11 (2.8%) samples contained *S. pombe* and *S. octosporus*. In the only sample from which *S. osmophilus* was isolated, *S. octosporus* was detected too.

**Fig. 3:**
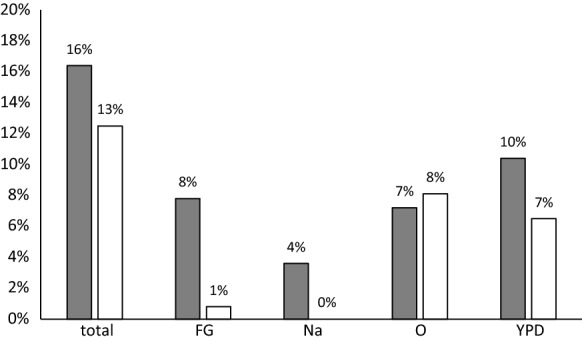
The success rate of different media used for isolation of *S. octosporus* (white) and *S. pombe* (grey) from honey

The success rate of the different enrichment media used for isolation is given in Fig. 3. Use of medium CY did not result in any yeast isolations. From all samples from which *S. octosporus* was isolated by the use of medium Na, *S. octosporus* was also isolated by the use of other media. The use of none of the other enrichment media alone would have led to the isolation of all *S. octosporus* and all *S. pombe* strains. From 6 samples, *S. octosporus* was isolated exclusively by use of medium O, from 9 samples it was isolated solely by use of medium FG and from 17 samples it was isolated only by use of medium Y. In these cases, the other media did not result in isolation of *S. octosporus*. From all other samples, it was isolated by the use of more than one medium. *S. pombe* was isolated from 17 samples by use of medium Y while the other media did not lead to the isolation of *S. pombe*. Further, it was isolated from 22 samples by use of medium O only. From these 22 samples *S. pombe* was not isolated using the other media.

**Fig. 4:**
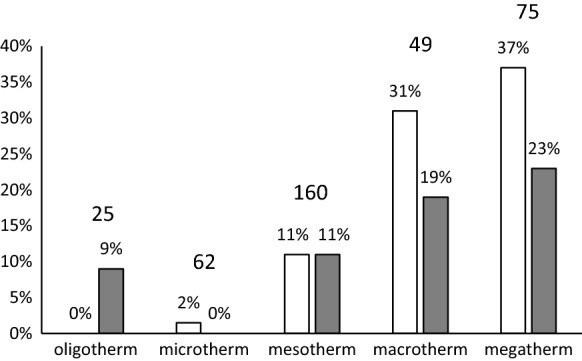
Frequency [%] of *S. octosporus* (white) and *S. pombe* (grey) in honey samples as a function of the isothermomene category from which the honey originates. The number of samples is given above the columns

The honey samples were assigned to the isothermomene (see “Discussion” section) as defined by Lauer et al. (1996) and Breckle and Rafiqpoor (2019). The frequency of *S. octosporus* and *S. pombe* in the different isothermomene is given in Fig. 4.

### Beehive materials

At total of 37 comb samples were investigated including both honey combs and combs with bee bread. Only one strain of *S. pombe* was found in a sample taken from a British beehive.

### Raw fermented cacao beans, nibs and powder

*Schizosaccharomyces pombe* was isolated from 50 (66.7%) out of 75 cacao product samples. 38 (71.2%) of 53 samples of cacao beans and 10 (47.2%) of 21 samples of cacao nibs harbored *S. pombe*. Only one sample of raw cacao powder was investigated. It contained *S. pombe*. From the same samples, with YPD medium 32 strains were isolated and with medium MC 46 strains were isolated. In 4 cases, use of YPD medium yielded *S. pombe* and use of MC medium did not. The other way around, from 18 samples, *S. pombe* was isolated when the enrichment was done with medium MC and not when enrichment was done with YPD medium. In many cases, during enrichment with YPD medium, budding yeasts developed. For arbitrarily selected strains of the budding yeasts the D1/D2 domain sequenced and analyzed. The D1/D2 domain of all strains tested (n = 13) were consistent with the D1/D2 sequence of the type strain of *Sa. cerevisiae*.

### Fermented tea

10 liquid starter cultures were investigated. By microscopic analysis of the starter cultures, no fission yeast cells were found. After enrichment of aliquots with medium MC, one starter culture yielded *S. pombe.*

## Discussion

### Isolation methods

Generally, an enrichment medium needs to permit growth of the target species and suppress or at least considerably slow down the propagation of other yeasts, molds and bacteria that may be present in a substrate that is investigated. Therefore, the effectiveness of an enrichment medium does not only depend on the characteristics of the target organism but on the characteristics of the co-occurring microorganisms too. As the co-occurring microorganisms may fundamentally vary from one substrate to another it is often unavoidable to use different enrichment media for different substrates even if one aims on isolating the same organism. Substrate properties such as pH, buffer capacity, osmotic pressure or salt contents may also affect the effectiveness of isolation media. Other factors like the way the samples are taken and treated or the incubation temperature and the duration of incubation may have great impact on the species isolated.

### *Schizosaccharomyces pombe*

As the few reports about the isolation of *S. pombe* in temperate climate all were related to different fruit, fruit fermentations (Chalenko and Korsakova 1961; Kudrjawzew 1960; Rzedowski and Rzedowska 1960) and soil under fruit-bearing plants (Davenport 1974), one of the main challenges in the current study was to develop a medium that would allow the targeted isolation of *S. pombe* from these substrates with an acceptable success rate.

*Schizosaccharomyces pombe* possesses several physiological characteristics that offer opportunities for the formulation of an enrichment medium being specific for *S. pombe.* These characteristics are the growth in the presence of 0.001% cycloheximide, high tolerance to weak organic acids like sorbic acid, tolerance to common SO_2_ concentrations as used in wine making, growth in 1% acetic acid or in high ethanol concentrations. Additionally*, S. pombe* grows at 37 °C, low a_w_ (water activity) and low pH values (Benito et al. 2013; Taillandier 1990; Vaughan-Martini and Martini 2011; Warth 1985; Whiffen 1948).

As *S. pombe* ferments at much higher SO_2_ concentrations than many other yeast (Maugenet and Escaré 1983; Osterwalder 1924; Pitt et al. 2009; Taillandier 1990; Warth 1985; Yang 1975) it seemed promising to use SO_2_ as a selective agent for *S. pombe*. Our results reveal that *Sac. ludwigii* and/or *Z. bailii* almost always occur on fruit and in fruit related soils. Both species are known to tolerate even higher concentrations of SO_2_ than *S. pombe* (Deak 2008; Rankine and Pilone 1973; Stratford et al. 1987; Warth 1985). While *S. pombe* was found to anaerobically grow in the presence of 122 mg/l free SO_2_ at pH 3.5 and 25 °C *Sac. ludwigii* and *Z. bailii* were shown to tolerate about 1.5 times more free SO_2_ (179 g/l) under the same conditions (Warth 1985). The inhibitory effect of SO_2_ ends when all free SO_2_ is bound by fermentation byproducts such as acetaldehyde and keto acids. After the free SO_2_ is bound, growth and fermentation take place unhampered. In the current investigation, *Sac. ludwigii* and/or *Z. bailii* were the dominant species in the sulfited grape and apple mash samples. *Schizosaccharomyces* cells were never observed.

In contrast to the findings reported here, in wine making *S. pombe* was repeatedly found to be the causative organism during unwanted fermentation of must that was treated with SO_2_ in order to preserve it for later use (Brugirard and Roques 1972; Castelli and Haznedari 1968; Delteil 1988; Maugenet and Escaré 1983; Osterwalder 1924; Taillandier 1990).

Florenzano et al. (1977) and van Zyl and du Plessis (1961) used the combination of SO_2_ with cycloheximide successfully for the isolation of *S. pombe* in Sicily and South Africa, respectively. Like numerous other yeasts *Sac. ludwigii* and *Z. bailii* do not grow in the presence of 0.001% cycloheximide (Whiffen 1948) whereas *S. pombe* does (Brysch-Herzberg et al. 2019; Whiffen 1948) we adopted the use of cycloheximide in medium CY (see below) for enrichment of *S. pombe*. Despite the positive reports we did not perform any more trials with SO_2_ because of its inactivation by yeast metabolites. Instead we tested other inhibitory agents such as benzoic acid, ethanol and acetic acid which all permanently suppress the growth of many yeasts during incubation and to which *S. pombe* is highly tolerant (Benito et al. 2013).

Many yeasts do not grow in the presence of sorbic acids at low pH values (Deak 2008). The tolerance of *S. pombe* to sorbic acid was described to be about as high as the tolerance of *Sac. ludwigii* and *Z. bailii* to this agent (Deak 2008; Warth 1985). *Sac. ludwigii* and *Z. bailii* are reported to be highly susceptible to cycloheximide (Benito et al. 2013). For these reasons we tested the combination of sorbic acid and cycloheximide for its aptitude to isolate *S. pombe*. We used the medium described by Benito et al. (2013). Although Benito et al. (2013) used the medium with good success for the isolation of *S. pombe* from honey the fast development of different yeasts and filamentous fungi in the fruit and soil samples incubated with this medium made clear that it is not adequate for these substrates.

From large scale isolation trials done before (Brysch-Herzberg and Seidel 2017) it was clear that 8% ethanol (medium O) without any other inhibiting agent would promote the growth of other species like *Sac. uvarum, Sac. paradoxus, Sac. cerevisiae, Lachancea thermotolerance* etc. in forest, fresh fruit or soil samples. In 283 samples incubated in medium O the authors did not detect *S. pombe*. Only in the case of substrates in which the dominance of the former species was not clear right from the beginning, eg. honey or dried fruit, medium O was used.

For the reasons given above, acetic acid was added to which *Saccharomyces* species and many other yeasts do not have a high tolerance. *S. pombe* grows in the presence of 1% acetic acid (Brysch-Herzberg et al. 2019; Pitt et al. 2009) and tolerates ethanol up to 8% (Vaughan-Martini and Martini 2011). Therefore, a medium (medium EE) was composed which contained 0.8% acetic acid and 6% ethanol. Pretests had shown that the combination of ethanol and acetic acid at these concentrations were about the maximum at which different strains of *S. pombe* were able to grow. Use of medium EE led to the isolation of *S. pombe* but the predominance of *Sac. ludwigii* and/or *Z. bailii* in many enrichments made it necessary to add an agent that would suppress their growth.

Because of the positive reports by Florenzano et al. (1977) and van Zyl and du Plessis (1961) (see above) cycloheximide at a concentration of 0.001% was added to medium EE resulting in medium CY. The high susceptibility of *Z. bailii* and *Sa. ludwigii* to this agent was previously reported (Benito et al. 2013). Benito et al. (2013) used 0.002% cycloheximide for the isolation of *S. pombe* from honey and Whiffen (1948) reported tolerance of *S. pombe* to 0.0025% cycloheximide. Brysch-Herzberg et al. (2019) investigated 13 *S. pombe* strains from all over the world and from different substrates which all grew in the presence of 0.001% cycloheximide. Although it cannot be excluded that *S. pombe* strains exist that are not able to grow in the presence of 0.001% cycloheximide, the results of the literature cited suggest that the majority of strains would grow. The faint gas formation observed in some samples in the first 2–3 days of incubation in medium CY most likely was caused by microorganisms that were susceptible to the medium but were not instantly deactivated. The very low number of false positive samples (< 0.5%) combined with the acceptable success rate of about 20% for the isolation of *S. pombe* from grape mash samples make CY-medium suitable for large scale isolation trials.

Not all strains of *S. pombe* are able to grow in the presence of 60% glucose (Vaughan-Martini and Martini 2011). Therefore, in the case of dried fruit medium O (8% ethanol) and in the case of honey media O and YPD were used in addition to medium FG. In the case of dried fruit medium O turned out to be effective. For the enrichment of *S. pombe* from honey, media O and YPD proved to be well suited. Omitting one of the media would have resulted in the loss of a considerable number of strains. Remarkable is that the success rate for the isolation of *S. pombe* was higher when the strongly restrictive medium O (8% ethanol) was used. Obviously, in the presence of 8% ethanol most strains of *S. pombe* were stronger competitors than the co-occuring strains of other yeast species in the samples. It is not clear why the use of medium CY for honey suppressed the growth of all *S. pombe* strains while its use was highly effective in the case of grape mash and rotting apples. Most likely the latter substrates contain substances that bind some of the inhibitors, e.g. parts of the acetic acid in medium CY could be bound by the potassium present in the fruit.

Following the instructions of Lilienfeld (von Lilienfeld-Toal 1927) the first approach to enrich *S. pombe* from cacao beans was to incubate the beans in a rich medium at 37 °C. While Lilienfeld-Toal (1927) used lactic acid in order to suppress bacteria we added chloramphenicol. Surprisingly, in many samples *S. cerevisiae* developed at this temperature besides *S. pombe*. For this reason, cycloheximide was added which suppresses the growth of *S. cerevisiae* (medium MC). Because growth in the presence of cycloheximide is more demanding the incubation temperature was lowered to 28 °C.

Both enrichment media used in the current study are well suited for the isolation of *S. pombe*. Both media have advantages and disadvantages. When the enrichment was done in YPD medium at 37 °C, budding yeasts often dominated. In some cases, the number of *S. pombe* cells was so low that it was not possible to isolate *S. pombe*. Although the samples were covered with oil, molds sometimes grew in the enrichment medium. After streaking such an enrichment medium on agar, the molds often covered the agar faster than *S. pombe* colonies developed. As a result, *S. pombe* could not be isolated from these samples. The advantage of YPD medium was that fermentation usually started within the first days and that incubation longer than one week never resulted in additional isolations of *S. pombe*. Hence, if isolation of *S. pombe* is possible by means of YPD medium, it usually can be done within one week. Incubation in MC medium needs to be done for 2 months because development of *S. pombe* in the medium can take such long times. The advantage of MC medium is that neither budding yeasts nor filamentous fungi developed in the medium.

### *Schizosaccharomyces octosporus*

One of the outstanding physiological characteristics of *S. octosporus* is its pronounced osmotolerance. It grows in the presence of 60% glucose (Barnett et al. 2000). Therefore, medium FG characterized by 60% sugar (5% glucose + 55% fructose) was used for the isolation of *S. octosporus*. Surprisingly, we recognized that *S. octosporus* was often fermenting honey samples and dried fruit samples that were incubated in medium O which contains 8% ethanol. Medium O was used with the intention to enrich *S. pombe*. As we knew from own oenological trials (data not shown) that *S. octosporus* is a much weaker fermenter than *S. pombe* we did not expect that an enrichment medium with such a high ethanol content could be successfully used for the isolation of *S. octosporus*. In the case of honey the dilution of the substrate with YPD medium was also often effective for the isolation of *S. octosporus*. Thus, aside from medium FG, media O and YPD were used for the isolation of *S. octosporus*. The first 192 honey samples were incubated in enrichment medium Na (16% NaCl). Because only a small number (n = 7) of isolations were done by means of this medium and because isolations were only done from honey samples from which *S. octosporus* was isolated with one of the other media we ceased use of this medium. As we isolated obligate osmophilic strains of *S. octosporus* (data not shown) it is important to streak the fermenting enrichment medium on a high osmotic agar (e.g. 30% glucose) and not on conventional YPD agar.

### *Schizosaccharomyces osmophilus*

*Schizosaccharomyces osmophilus* does not grow well or does not grow at all in and on low osmotic media (Brysch-Herzberg et al. 2019). In the current study it exclusively occurred in samples incubated in medium FG. For isolation of *S. osmophilus* it is crucial to streak the fermenting enrichment medium on high osmotic agar.

### *Schizosaccharomyces japonicus*

Besides its tolerance to ethanol, we are not aware that *S. japonicus* would possess any other marked tolerance against inhibitors like acetic acid or cycloheximide. Additionally, it does not grow at extreme temperatures or at high osmotic pressure (Barnett et al. 2000). Therefore, we did not succeed in developing a more selective isolation medium or incubation conditions for *S. japonicus* than the use of medium O. As we know from our own data, *S. japonicus* develops in this medium in the presence of different *Saccharomyces* species that usually dominate in field samples incubated in medium O (Brysch-Herzberg and Seidel 2017).

### Ecology

#### *Schizosaccharomyces pombe*

Of the 161 *S. pombe* strains that were included in a population genomic study by Jeffares et al. (2015) the great majority was isolated from substrates which are the result of human activity and which do not occur in nature. The authors obtained most of the strains from culture collections. The most important substrates in their investigation were molasses or sugar cane juice fermentations for the production of rum and cachaça, cacao and coffee bean fermentations or grape must fermentations for wine making. Fittingly, 55 of the 57 *S. pombe* strains held by the Westerdijk Fungal Biodiversity Institute, Utrecht, The Netherlands were isolated from food, beverages or substrates related to food and beverage production (Personal communication with Marizeth Groenewald, curator of the yeast collection at Westerdijk Fungal Biodiversity Institute).

In the past *S. pombe* was sporadically isolated from wine grapes, grape must and grape wines (Florenzano et al. 1977; van Zyl and du Plessis 1961), vineyard soil (Davenport 1969), fresh fruit other than grape berries, their musts and wine made thereof (Kudrjawzew 1960), honey (Benito et al. 2013), sugar cane molasses (Alan 1906), beer (Lindner 1893), palmwine (Amehd et al. 1994), cacao beans (von Lilienfeld-Toal 1927), tea fermentations (Reiß 1987) or coffee bean fermentation (de Melo Pereira et al. 2012).

In the current investigations, grape wine related substrates, fresh and dried fruit of different kind, commercial honey as well as bee hive materials, solitary bee beebread, raw (unroasted) cacao and raw coffee beans, sugar beet molasses, tea fermentation starter cultures and different forest materials were investigated.

In the following the results obtained in the current study are discussed in the context of various literature reports. The results of each culture based study are tremendously influenced by the sampling and isolation methods. Therefore, the methods described in the literature are considered wherever this is possible.

#### Occurrence of *S. pombe* on grape wine related substrates

For a better understanding of the results obtained by various authors the different materials in the wine making process are briefly explained here: Commonly, after harvest the first steps of wine production from grapes is destemming and crushing of the grape material. Often, in the case of white wine production the resulting mash is allowed to rest for a certain time (up to 24 h) before the mash is pressed and the raw turbid must, after a clarification step, is fermented to wine spontaneously or after the addition of pure yeast culture material. For the production of red wine the pulp is directly fermented. In this case pressing usually takes place after completion of the fermentation process. Thus, the materials turbid must in the case of white wine production and the fresh unfermented pulp in the case of red wine production can be seen as comparable materials as they are the last stage before fermentation. Potentially they should harbor about the same spectrum of yeasts species. Naturally, the density of yeast cells should be significantly higher in grape mash as it still contains all solid matters of the harvested grapes except the stems.

In the current study, the presence of *S. pombe* on grapes collected in the vineyard, grape mash from different wine producing facilities as well as vineyard soils was examined in the wine growing area Württemberg in southwest Germany.

#### Grapes, other vineyard materials and grape mash

In 2017 and 2020 about 13% and 21% of the grape mash samples harbored *S. pombe*, respectively. *S. pombe* was not isolated from grapes directly collected in the vineyard and not from vineyard soil. In Germany, in an earlier investigation, the yeast communities on grapes was investigated by a direct plating approach. *S. pombe* was not detected in this study, too (Brysch-Herzberg and Seidel 2015). In a comparable climate in Great Britain, Davenport (1969, 1971, 1974, 1975) isolated *Schizosaccharomyces* spec. from vineyard phylosphere and rhizosphere applying an enrichment procedure. Davenport (1971) solely isolated *Schizosaccharomyces* spec. from acid but not from alkaline vineyard soil. However, in agreement with the current results, *Schizosacharomyces* species were very rare in this investigation.

In South Africa, similar results were obtained by van Zyl and du Plessis (1961) who made a large scale investigation on the occurrence of yeasts in wine and various substrates related to the wine making process. The authors did not find *S. pombe* in a total of 81 samples from vineyard environments consisting of vine bark, vine pedicels and vine flowers as well as ripe and unripe wine grape berries. The authors did not perform any enrichment step. Other studies performed in South Africa confirmed that *S. pombe* is usually not isolated by direct plating techniques without any enrichment step: Bagheri et al. (2015) monitored the yeast population of three neighboring vineyards under different viticulture regimes by direct plating of grape must. In another study conducted in South Africa over 3 vintages Jolly et al. (2003) investigated the yeast communities in samples originating from 4 different vineyards. Isolation was done by direct plating. By automated ribosomal intergenic spacer analysis (ARISA) and by direct plating of wash solution from undamaged berries, Setati et al. (2012) did not detect *S. pombe* in three adjacent vineyards in Stellenbosch.

In Italy, special attention was paid to the occurrence of *S. pombe* on wine grapes and wine related substrates by several authors: The presence of *S. pombe* on grapes, in musts and resulting wines was shown in several investigations in Italy (Balloni et al. 1970; Delfini 1985; Delfini et al. 1983; Florenzano 1972; Florenzano et al. 1977; Messini et al. 1985) and on Malta (Amerine and Kunkee 1968). In Northern Italy, during the harvest in 1983, *S. pombe* occurred as spoiling organism in many fermentations. As a source of *S. pombe*, the authors identified grapes particularly rotting grapes (Delfini 1985). By means of an enrichment medium based on the selective effects of cycloheximide and SO_2_, Florenzano et al. (1977) were able to show that *S. pombe* is a common member of the yeast communities on Sicilian grapes. They isolated *S. pombe* from 26 out of 68 grape samples. The authors concluded that in other investigations the failure to detect *S. pombe* on grapes most likely is a consequence of inadequate isolation methods lacking an effective enrichment procedure.

In other countries, *S. pombe* was isolated from grapes, parts of vines or vineyard soils: e.g. Venezuela (Araujo et al. 1998; Balloni et al. 1966), Ukraine (Bayraktar 2014), Spain (Rankine and Fornachon 1964) and India (Relan and Vyas 1971). Araujo et al. (1998) and Bayraktar (2014) isolated *S. pombe* by means of enrichments. The other authors did not specify their isolation technique.

Although in the current study *S. pombe* was isolated successfully from grape mash we were not able to isolate it directly from soil or grape samples taken in the vineyard. These results are in compliance with the great majority of studies from all over the world in which *S. pombe* was not detected in vineyard substrates. *S. pombe* was not detected e.g. in California (USA) (Mrak and McClung 1940), Chile (Ganga and Martínez 2004; Jara et al. 2016), Argentina (Combina et al. 2005), Brazil (Bezerra-Bussoli et al. 2013), New Zealand (Zhang et al. 2010), Australia (Prakitchaiwattana et al. 2004), China (Li et al. 2010; Sun et al. 2014), India (Chavan et al. 2009), Israel (Zahavi et al. 2002), Sicily (Romancino et al. 2008), Slovenia (Raspor et al. 2006), Switzerland (Díaz et al. 2013) and Spain (Clavijo et al. 2010).

Based on our own results and on the information derived from the literature, we conclude that although *S. pombe* can be isolated from grapes and from vineyard soil if an adequate enrichment medium is used and numerous samples are investigated. It is neither as frequent nor as abundant as many other yeast species in these substrates. The fact that *S. pombe* was found in 13% to 21% of the grape mash samples in the current study may at first glance be contradictory to this conclusion but the relatively high percentage of samples harboring *S. pombe* may be misleading for several reasons: First, the grape mash in the wine producing facilities can be seen as a blended samples consisting of thousands of grape bunches with a total volume of up to several thousand liters per grape delivery. If only one grape bunch is heavily infected by *S. pombe*, it would easily be enough to infect the whole grape mash produced from one grape delivery. The second reason why *S. pombe* was isolated from more than one tenth of the grape mash samples may be the effectiveness of the enrichment medium CY. The medium seems to be highly effective in suppressing all other microbes but allowing *S. pombe* to develop even from a very few cells. Additionally, it is possible that *S. pombe* cells from the preceding grape delivery were still present in the tubes and pumps and thus detected in the following grape delivery.

#### Must, must fermentations, grape mash fermentations and wine

van Zyl and du Plessis (1961) investigated 25 unfermented and 92 fermented grape must samples. Unfermented must did not yield any *S. pombe* isolates while *S. pombe* was isolated from 10 fermented must samples (12% of all must samples). Only in case the samples were treated with cycloheximide *S. pombe* predominated after fermentation.

In North Italy in 1983 a total elimination of malic acid occurred in musts during fermentation. As the causative organism *Schizosaccharomyces pombe* was identified (Delfini et al. 1983). *S. pombe* was detected in wine fermentations in Spain (Torija et al. 2001), in Greece (Jörgensen 1909), South Africa (van der Walt and van Kerken 1958) and Portugal (Couto et al. 2005). In Argentina *S. pombe* was detected in several samples of concentrated grape juices (Combina et al. 2008). It was isolated from fermenting grape must that had been conserved with high concentrations of sulfur from Malta (Castelli and Haznedari 1968) and from France (Brugirard and Roques 1972; Castelli and Haznedari 1968; Delteil 1988; Maugenet and Escare 1983; Osterwalder 1924; Taillandier 1990). In these cases, the preserved must seems to act as a highly selective enrichment medium that only allows a very few specialized yeasts to develop such *as Sa. ludwigii, Z. bailii* and *S. pombe* (Warth 1985).

In various investigations in countries like China (Sun et al. 2014), Czech-Republic, Slovakia, Hungary (Minarik 1964), Slovenia (Jemec et al. 2001), Austria (Lopandic et al. 2008), Germany (Sturm et al. 2006), Switzerland (Díaz et al. 2013; Schütz and Gafner 1993), Italy (Tofalo et al. 2009), France (Fleet et al. 1984; Zott et al. 2008), Spain (Beltran et al. 2002; Clemente-Jimenez et al. 2004; Hierro et al. 2006), Portugal (Barata et al. 2008; Couto et al. 2005), USA (Egli et al. 1998; van Keulen et al. 2003), Brazil (Bezerra-Bussoli et al. 2013) and Australia (Heard and Fleet 1985), *S. pombe* was not detected. Most likely this is due to the fact that *S. pombe* is a week competitor against other indigenous yeast in spontaneous grape must fermentations or against inoculated starter cultures (Brugirard and Roques 1972; Delteil 1988; Ribereau-Gayon and Peynaud 1962).

As in the case of vineyard materials the examples given above illustrate that it is an exception if *S. pombe* is detected in musts or fermenting musts. Without a selective agent such as cycloheximide or SO_2_ it is hardly can be isolated at all which can be interpreted as a strong hint that *S. pombe* is present infrequently and with low cell numbers in the wine production process compared to other species such as *Hanseniaspora uvarum, Sac. cerevisiae* or *Pichia membranifaciens*.

#### Wine

In 200 of 480 wines, budding yeasts were detected but only one wine contained *S. pombe* (van Zyl and du Plessis 1961). The low incidence in wine is in agreement with the findings of van der Walt and van Kerken (1958) who examined 60 turbid wines containing yeasts and did not detect *S. pombe* at all. Frequently *S. pombe* is described as a wine spoilage yeast. Although *S. pombe* has the potential to fermented residual sugar in wines and spoil the product, we never found it in hundreds of wines we have routinely investigated by membrane filtration in our laboratory (data not shown). Strain CBS 2629 (*S. japonicus*) was isolated from Portuguese white wine and CBS 10500, 10501, 10502 from South African wine. At least today, when effective membrane filter techniques have become a standard in all wine producing facilities, *S. pombe* does not seem to be a common contamination in wines.

#### Apples

*Schizosaccharomyces pombe* strain CBS 5680 was isolated from apples in Poland. Similarly, the presence of *S. pombe* on apple or in fermenting apple must in temperate climate was reported (Davenport 1969; Rzedowski and Rzedowska 1960). Therefore, in the current study apples collected in apple plantings, the soil of apple plantings and fresh apple musts were investigated. Only one sample of each substrate harbored *S. pombe* although in the case of soil and fresh apple both collected under apple trees a considerable number of samples was investigated. It seems remarkable that the investigation of only 6 apple must samples led to the isolation of *S. pombe*. As in the case of grape mash apple must can be seen as a blended sample of thousands of apples. This may be the reason why the isolation result of 6 must samples was the same as in 40 apple samples.

During harvest and during storage, commercial apple growers need to sort out huge amounts of substandard, injured or rotting apples. Most apple growers have dumping sites where they compost these apples. Often these sites are used for years. 42% of the rotting apple samples from such dumping sites contained *S. pombe.* In Germany *S. pombe* was detected at each site and in China at 1 of 2 sites. The smell of the fermenting mounds of apples had a distinct note of acetic acid and alcohol. Under the microscope the characteristic cells of *Sc. ludwigii*, which tolerates high concentrations of both agents, were omnipresent. Thus it could be assumed that the mounds of rotting apple function as an enrichment environment not only for *Sc. ludwigii* but for *S. pombe* too. The species that often dominate under fermentative conditions such as S*ac. cerevisiae, Sac. uvarum, Sac. paradoxus* or *Lachancea thermotolerance* could be suppressed by acetic acid which could give an advantage to *S. pombe*. In this way the initial presence of a few *S. pombe* cells on a few individual apples could lead to significant populations in the mounds of rotting apples.

#### Other fresh fruit and fruit fermentations

Besides wine grapes and apples, *S. pombe* was detected in connection with other fresh fruit and fruit must fermentations in earlier investigations. Repeatedly, different authors mention the presence of *S. pombe* in fruit wine fermentations (Chalenko and Korsakova 1961; Kudrjawzew 1960; Rzedowski and Rzedowska 1960), Additionally, *S. pombe* was detected on rotting papayas (Maragatham and Panneerselvam 2011), on rotting mangos (Somda et al. 2016) and on ripe plums of different varieties and origins in Argentina (de Erezcano de Migoya 1953).

Our own results and the various reports in the literature leave no doubt that *S. pombe* is regularly present on fresh fruit. Our findings confirm the conclusions of Florenzano et al. (1977) who highlighted the necessity of an effective enrichment medium in order to study the occurrence of *S. pombe*. Direct plating of fruit must or wash solution of the fruit does usually not lead to success (see above). As given above, in the current study *S. pombe* was frequently isolated only by means of a highly specific enrichment medium from blended material of grapes and apples consisting of many individual fruit (grape mash and piles of rotting apple). When the fruit were collected in the field and only 3–4 apples or 1–2 grape bunches were used as one sample and an aliquot the material was incubated in the enrichment medium only a single isolate was retrieved from 35 apple and none from 30 grape samples. Thus, it can be concluded that *S. pombe* is present on a very few individual fruit only unlike other yeasts such as *Hanseniasporum uvarum* or *Metschnikowia pulcherrima* which are omnipresent on fruit. As *S. pombe* is such rare on individual fruit and large fruit accumulations comparable to the fermenting apple piles of apple farmers do not exist in nature it seems questionable that fruit are the primary habitat of *S. pombe*. On the other hand, the high tolerance of *S. pombe* to acetic acid, to alcohol and low pH-values suggest that fermentation plays an important role in *S. pombe*’s live history. In this context the ability of *S. pombe* to ferment malic acid to alcohol could be interpreted as an adaptation to low pH fruit habitats. The maloalcoholic fermentation raises the pH which could make it easier for *S. pombe* to proliferate.

#### Dried fruit

In the current study *S. pombe* was frequently isolated from mango (~ 30%) and pineapple samples (~ 23%). From raisin samples it was isolated comparatively infrequent (~ 10%). *S. pombe* was isolated from only 1 out of 13 papaya samples and 1 out of 3 kaki samples. In the case of kaki the low number of samples available for investigation demand further investigation before *S. pombe*’s frequency on the fruit can be assessed. Our findings are in agreement of those reported by Tokuoka (1993) who isolated *S. pombe* from dried fruit namely from raisins (Tokuoka et al. 1985) before.

From samples of dried figs (n = 14), plums (n = 21), dates (n = 29), apricots (n = 15), apples (n = 10) and cherries (n = 16) *S. pombe* was not isolated at all. Although apricots, dates and raisins are sometimes grown in the same subtropical country, e.g. Turkey, there is a great difference between the different fruit. The same is true for mango, pineapple and papaya which are all grown in the same tropical countries. Even though the results indicate differences between the fruit grown I n one area it is not possible to judge if the differences are due to the nature of the fruit themselves, the way the fruit are dried or the kind of animal (insects) that visits the fresh and/or drying fruit. For this reason, it is not possible to guess why Phaff et al. (1966) isolated *S. pombe* repeatedly from dried figs, dates and prunes whereas we did not.

Dried fruit may occur without targeted drying of fruit, as fruit mummies on different kind of fruit-bearing plants. Sipiczki (2016) showed for typical wine yeasts overwinter with grape mummies in vineyards in Slovakia and Hungary. Because of its low frequencies and assumed low cell numbers on fresh fruit as apples and grapes it seems questionable if *S. pombe* overwinters on fruit mummies.

#### Honey

The overall frequency of *S. pombe* in honeybee honey was 12.7%. The overall frequency can be misleading because the frequency varied significantly between countries. While in some countries with tropical and subtropical climate its frequency was about 25% *S. pombe* was rarely found in boreal or temperate climate. For example, in Finnish honey (n = 16) *S. pombe* was isolated from one sample only. In Germany it was detected in 5% of the honey samples (n = 106). In Spanish honey (n = 38) and Mexican honey (n = 22) it was detected with a frequency of 23.7% and 27.3%, respectively.

As the frequencies in different countries having different climate suggested that *S. pombe* was more frequent in honeys coming from warmer regions, each honey sample was assigned to an isothermomene category as defined by Breckle and Rafiqpoor (2019), Lauer et al. (1996). Isothermomene are lines of equal thermal vegetation length. The thermal vegetation length is the period of the year in which the temperature theoretically allows the plants of a certain zone to grow. Other factors like water availability, sun radiation or late frost events are not considered (Breckle and Rafiqpoor 2019; Lauer et al. 1996). The frequency of *S. pombe* together with that of *S. octosporus* in dependency of the isothermomene is illustrated in Fig. 4.

Statistical evaluation of the differences between the frequencies of *S. pombe* in honey samples from different isothermomene categories was done with the Kruskal-Willis H Test. The differences were not statistical significant. From the coldest isothermomene category (oligotherm) 25 honey samples were investigated. One from Norway and one from Finland contained *S. pombe* while none contained *S. octosporus*. Data of more honey samples from the cold boreal regions are required, before it can be assessed if *S. pombe* actually is more frequent in the cold regions than *S. octosporus*.

Reports about the presence of *S. pombe* in honey are rare. While it was not detected in honeys from Portugal (Carvalho et al. 2005, 2010), Thailand (Saksinchai et al. 2012), Japan (Furuta and Okimoto 1978), and the USA (Fabian and Quinet 1928) its presence in Spanish (Benito et al. 2013) and Sudanese honey (Abubaker et al. 2012) was reported. Compared to other species which we isolated more often from honey like *Z. rouxii*, *Z. mellis* (data not shown) or *S. octosporus, S. pombe* possesses a moderate osmotolerance. Only a subset of strains is able to grow on 60% glucose (Vaughan-Martini and Martini 2011) which is equivalent to an a_w_ of about 0.85. Water activity in most honeys ranges between 0.50 and 0.67 (Kačániová et al. 2021; Zamora and Chirife 2006). Thus, in ripe honey *S. pombe* is not able to proliferate. Additionally, *S. pombe* is not commonly found in floral nectar (Brysch-Herzberg 2004) which otherwise could have explained the regular occurrence of *S. pombe* in honey. Nonetheless, as discussed in detail below honey may either temporarily or just in a few single combs offer an adequate substrate for proliferation for *S. pombe*.

Despite the properties of ripe honey given above *S. pombe* occurs regularly in honey. In connection with honey bee honey the ability of *S. pombe* to grow and ferment in the presence of high alcohol and acetic acid concentrations as well as its moderate osmotolerance could be of advantage. Fermentation in honey bee honey occurs rather frequently in the combs (Mccleskey and Oertel 1950). Specially in low concentrated unripe fermenting honey (see “Discussion” section below for *S. octosporus* in honey) *S. pombe* could at least temporarily be a strong competitor.

#### Beehive materials

Only one strain of *S. pombe* was found in a sample taken from a British beehive although a total of 36 samples was investigated. This finding is consistent with the results obtained from honey. *S. pombe* can be isolated from honey in temperate zones but it is not frequent. *Schizosaccharomyces sp. were* found in the crop of honey bees by Batra et al. (1973). Therefore, their presence in beehives seems just consequent.

#### Substrates related to sugar production and spirit production from sugar cane

In the literature, different distillated alcoholic beverages produced from sugar cane are mentioned in connection with the occurrence of *S. pombe*, repeatedly. Therefore, a very short overview of the different types of distillates is provided here: Sugar cane is used for the production of alcoholic beverages in all tropical regions of the world where it is cultivated. In the Caribbean, Rum probably being the most well-known product is made by the fermentation of molasses, syrup or fresh sugar cane juice. Molasses are the residuals of sugar production from which sucrose cannot be further obtained and syrup is produced by vacuum distillation from cane juice. Molasses usually contain some 50–60% sugar (Lethonen and Suomalainen 1977). Cachaça, Aguardente de Cana and similar products are products which usually resulted from the fermentation of fresh sugar cane juice in South America, mainly Brazil. Arrack from Indonesia, China, Sri Lanka and neighboring countries is often made from sugar cane molasses or sugar cane juice but from other raw materials like rice or palm sap, too.

In the same year that Lindner (1893) described *S. pombe* from African millet beer, Vorderman (1893) found *S. pombe* in molasses fermentations in Yakarta, Indonesia, as can be seen undoubtedly from his drawings (Plate 2 of the original article). One year later this finding was confirmed by Eijkman (1894) who detected *S. pombe* in molasses fermentations for arrack production in Taiwan. The presence of *S. pombe* in molasses originating from Thailand, Japan and Taiwan was later reported by Tokuoka et al. (1985) who additionally isolated *S. octosporus* from his samples.

Early, the presence of *S. pombe* in the rum production process in Jamaica (Alan 1906; Ashby 1909; Greig 1895; Jörgensen 1909; Lund 1958), Guadeloupe, Martinique, Haiti and other Caribbean Islands (Fahrasmane et al. 1985; Parfait and Sabin 1975) was reported. Fahrasmane et al. (1988) isolated *S. pombe* from molasses, rum fermentations, soil of sugar cane plantings and soil around a rum manufacturing facility on Haiti. Additionally, *S. pombe* was found in 3 out of 10 sugar mills in Mexico (Bonilla‐Salinas et al. 1995). In a more recent study on the microbiology of rum production conducted in Queensland, Australia, *S. pombe* was isolated from 6 out of the 16 samples of molasses taken at the same rum producing facility before clarification of the raw material (Green 2015). In this investigation, *S. pombe* was the second most frequent species after *Sa. cerevisiae*. The relatively high fermentation temperatures of 28–37 °C (Lehtonen and Suomalainen 1977; Nicol 2003) are in favor of *S. pombe* that is tolerant to these temperatures (Vaughan-Martini and Martini 2011). Not in all investigations *S. pombe* was present in molasses (e.g. see the report of Hall et al. 1937).

In Brazil, cachaça (aguardente), a hard liquor made by fermentation of sugar cane juice followed by distillation, is produced. A short overview of the traditional preparation of the microbial inoculum for cachaça fermentation is given by Schwan et al. (2001) and Pataro et al. (1998). Usually, the fermentation is performed as repeated batch fermentation. One cycle usually takes 12–48 h. After one cycle, about 4/5 of the fermentation volume is removed for distillation and the fermentation vat is refilled with fresh sugar cane juice. A new inoculum is only used in the case of false fermentations resulting in off flavors or stuck fermentations (Schwan et al. 2001). In most investigations on the yeast communities of cachaça fermentations, *Saccharomyces* species are most frequent and most abundant. *S. pombe* occurs with high densities but irregularly: Shehata (1960) reported *S. pombe* to be one of the three most abundant species in sugar cane juice fermentation but only detected it in 2 of five factories investigated. Similar results were reported by Oliveira et al. (2004) who isolated *S. pombe* once during an investigation including 5 different distilleries. Gomes et al. (2002) succeeded in isolating 27 *S. pombe* strains which came from all seven cachaça distilleries investigated. The results of Pataro et al. (2000) may shed some light on why the study results vary such a lot. The authors investigated the yeast communities of sugar cane juice fermentations in three distilleries at three different times of the fermentation process during the harvest season. In one distillery they detected *S. pombe* after 31 days and in the other 2 distilleries it was only detected after 61 days of repeated batch fermentation. In light of these results, it is not surprising that Schwan et al. (2001) did not isolate any *S. pombe* strains although they investigated the yeast communities of 15 distilleries. In their investigation samples were taken 6–8 h after inoculation with the traditional self-made microbiological inoculum. If *S. pombe* has a tendency to occur in detectable cell densities late in the fermentation process, this could explain why it was not detected in several other investigations on cachaça fermentations (Morais et al. 1997; Silva et al. 2009), too. Additionally, Martini et al. (2010) did not find any *Schizosaccharomyces* yeasts in sugar cane juice and it was not detected in or on sugar cane itself (Khunnamwong et al. 2018; Nasanit et al. 2015; Srisuk et al. 2019). This suggests that *S. pombe* is present in low cell densities at the beginning of the fermentation process. The majority of *S. pombe* strains isolated from cachaça fermentations is able to ferment glucose at 41 °C and is able to adapt to alcohol concentrations of 10% (Gomes et al. 2002). Both values are about the maximum that would be expected to occur in cachaça fermentations (Gomes et al. 2002). Thus, these strains seem to be well adapted to the conditions of cachaça fermentations. Fittingly, *S. pombe* was detected in the bioethanol production plant which works on the basis of sugar cane sap (Cabrini and Gallo 1999). In Korea, *S. pombe* was isolated from the soil of an ethanol factory (Choi et al. 2010).

In our own investigation we did not detect S. pombe in 18 samples of German and Dutch sugar beet molasses. As we did not find any yeasts at all it could be that in the production process yeasts were destroyed by high temperatures. From the literature cited above, it can be concluded that in tropical countries *S. pombe* is regularly present in molasses and sugar cane juice. As described above the frequency with which *S. pombe* is detected seems to strongly depend on stage of the production process. It could very well be that *S. pombe* would be isolated with a higher success rate if an enrichment step in a medium containing 0.001% cycloheximide and 6% ethanol that would suppresses most budding yeasts and all molds was applied.

In cane sugar, *S. pombe* was detected too (Lodder and Kreger-van Rij 1952; Owen 1949). *Schizosaccharomyces pombe* strain CBS 10392 was isolated from raw cane sugar and *S. pombe* strains CBS 10503 and CBS 10504 were isolated from industrial glucose. In contrast, (Scarr 1951) did not isolate any *Schizosaccharomyces* strains from raw sugar and intermediate products.

#### Cacao

In 1909, Jörgensen (1909) reported the isolation of *S. pombe* from cacao beans in his laboratory in Copenhagen. The first global investigation was done by von Lilienfeld-Toal (1927) who investigated the yeast communities of cacao beans imported to Germany. He isolated *S. pombe* from beans coming from Ecuador, Venezuela, Brazil, Kameron, Gold Coast (Ghana), St. Thomé (India), Sri Lanka, Java and Trinidad. According to the author, *S. pombe* was one of the most frequently isolated yeasts from cacao beans. Isolation was done by an enrichment step in wart medium acidified with lactic acid. The presence of *S. pombe* in cacao bean fermentations and on fermented cacao beans was confirmed afterwards (Fowler et al. 1997; Lund 1958; Roelofsen 1958).

Later, *S. pombe* was detected in cacao fermentations in Mexico (Arana-Sánchez et al. 2015), Brazil (Illeghems et al. 2012), Trinidad (Rombouts 1952), Ivory Coast (Visintin et al. 2016), Ghana (Nielsen et al. 2007), Madagascar (Ravelomanana et al. 1984) and in Malaysia (Papalexandratou et al. 2013). In Nigeria, Faparusi (1974) isolated *S. pombe* from ripe cacao fruit, suggesting that initially *S. pombe* comes into the fermentations with the fruit.

*Schizosaccharomyces pombe* was not detected in cacao fermentations in Brazil (Camargo et al. 1963; da Veiga Moreira et al. 2013; Martelli and Dittmar 1961; Serra et al. 2019), in Ivory coast (Koffi et al. 2017; Koné et al. 2016), in Indonesia (Ardhana and Fleet 2003), in Ghana (Jespersen et al. 2005), in Ecuador (Papalexandratou et al. 2011), in Cuba (Maura et al. 2016), in Dominican Republic (Gálvez et al. 2007) and in Ivory Coast (Ravelomanana et al. 1985).

For some countries like Brazil or Ivory Coast, the results concerning the presence of *S. pome* are contradictory. While Visintin et al. (2016) found *S. pombe* in cacao fermentations in Ivory Coast, Ravelomanana et al. (1985) did not. This is the more remarkable as Ravelomanana et al. (1984) investigated cacao fermentations during 4 different years. This example suggests that other factors than the country of origin like the sampling scheme or the isolation technique may have a major impact on the detection of *S. pombe*. This conclusion is supported by the results presented here: In the current study, strains of *S. pombe* were isolated from commercial cacao products consisting of or produced from fermented and unroasted cacao beans. 2/3 of the samples yielded *S. pombe*. These came from 15 different countries. In the samples coming from Colombia and Sri Lanka (2 per country), we did not detect *S. pombe*. Most likely this is due to the low number of samples investigated.

In contrast to the reports cited above, we isolated *S. pombe* from samples originating from Indonesia, Ghana, Ecuador, Dominican Republic and Ivory Coast. In agreement with the assessment of von Lilienfeld-Toal (1927) and Rombouts (1953), we conclude that *S. pombe* is present in cacao fermentations worldwide. *S. pombe*’s high frequency of 66% in cocoa beans suggest that it is a typical member of the yeast communities of fermenting cacao beans.

During cacao bean fermentation the temperature rises fast. After 1–2 days it exceeds 40 °C at which the great majority of yeast species are not able to grow. *S. pombe* growth at 37 °C and some strains are even tolerant to 42 °C (Roelofsen 1958). In addition to the high temperature the concentration of acetic acid is about 1.2% after 39 h of fermentation and the ethanol concentration reaches a maximum of 3.5% in the liquid fraction. Because of its tolerance to these agents and because of its tolerance to high temperatures *S. pombe* seems to be well adapted to the conditions in cacao bean fermentation.

#### Coffee beans

*Schizosaccharomyces pombe* was detected in the fermentation of coffee beans in Brazil (Agate and Bhat 1966; Silva et al. 2000). We incubated 18 samples of coffee beans in media MC and YPD and did not detect *S. pombe*. We cannot say why but *S. pombe* seems to be much more frequent on cacao beans than it is on coffee beans.

#### Substrates related to the production of palm wine

In many countries of South America, Africa and Asia where palms grow, palm wine, often called “Toddy”, is produced from the palm sap. Different palm species are used for the production of palm wine. Palm sap is obtained by cutting parts of the palm or by incisions. A comprehensive overview of the production of palm wine is given by Okafor (1978). Palm wine is consumed fresh, pasteurized and bottled, or distilled. The distilled liquor is widely known as “Arrack”.

*Schizosaccharomyces pombe* was detected in many countries and regions of the world in freshly tapped sap or in fermenting palm sap e.g. in Pakistan (Ahmad et al. 1954), in Burkina Faso (Ouoba et al. 2012), in Nigeria (Amanchukwu et al. 1989; Boboye and Dayo-Owoyemi 2008; Faparusi and Bassir 1971; Obisanya et al. 1987; Sanni and Lönner 1993), in North Africa (Castelli and Haznedari 1968), in India (Shamala and Sreekantiah 1988), Sri Lanka (Atputharajah et al. 1986; Field and Wills 1998; Vidanapathirana et al. 1983) and Thailand (Udomsaksakul et al. 2018). *S. octosporus* was isolated from palm wine fermentation in Nigeria (Chilaka et al. 2010).

*Schizosaccharomyces pombe* was not found in every study in fresh palm sap or fermenting palm sap e.g. in the investigations of Yamagata et al. (1980) and (Stringini et al. 2009). Unfortunately, the authors of the current study did not have the chance to investigate fresh or fermenting palm sap. Nevertheless, the many reports of its presence in these substrates suggest that it is typical for its spontaneous fermentation. Based on our experience with the isolation of *S. pombe* and because most studies report the predominance of budding yeasts, an enrichment step in the presence of 6% alcohol and 0.001% cycloheximide could help to investigate *S. pomb*’s true frequency in palm sap fermentations.

#### Beer

Beer is produced in many countries of the world from different starch containing seeds such as corn (maize), rice, millet and different kind of grains. As it is well known, the strain on which Lindner based the species description of *S. pombe* was isolated from millet beer named “pombe” (Lindner 1893). Similarly, *S. pombe* strains CBS 5682 and CBS 10391 were isolated from African beer by J.P. van der Walt and S. James, respectively. Despite of these isolations, based on his own results and on reports of others, Van Der Walt (1956) states “The popular belief that the alcoholic fermentation of kaffir beer would be due only to the above mentioned organism (*S. pombe*, remark of the authors) seems to be unfounded”. This view is supported by studies on various types of traditional beer in different African countries in which *S. pombe* was not found (Demuyakor and Ohta 1991; Ekundayo 1969; Pattison et al. 1998). Fittingly, *S. pombe* was detected in only one out of ten samples of pito, a traditional beer, in Ghana by Sefa-Dedeh et al. (1999). Far away from Africa, strain CBS 1057 was isolated from brewer yeast in Skäne, Sweden. Morris and Eddy (1957) detected *S. octosporus* as a contamination of brewing “pitching yeasts”. Based on the cited study results, we conclude in line with Van Der Walt (1956) that undoubtedly *S. pombe* occurs in traditional beer fermentations but only in rare cases.

#### Fermented tea products

In a lot of countries black tea enriched with sugar is fermented with a “tea fungus”. The beverage produced by the fermentation is often called “Kombucha”. The “tea fungus” is a tough fibrous mass consisting of cellulose and acid producing bacteria and yeasts of different genera including *Schizosaccharomyces* (Greenwalt et al. 2000; Teoh et al. 2004). The presence of *S. pombe* in tea fermentations is reported by several authors (Herrera and Calderon-Villagomez 1989; Jankovic and Stojanovic 1994; Reiß 1987; Singh and Klar 2002; Teoh et al. 2004). Villarreal-Soto et al. (2020) identifies *S. pombe* as the dominant yeast in tea fermentations whereas other authors did not detect *S. pombe* at all (Marsh et al. 2014; Phaff et al. 1978).

We can confirm the presence of *S. pombe* in tea fermentation. The relatively low number of samples does not allow an estimate of its frequency.

#### Maotai-flavor Chinese liquor

*Schizosaccharomyces pombe* is reported to be a dominant and important yeast in the grain fermentation process for the production of Maotai-flavor liquor in China (Du et al. 2021; Song et al. 2017; Wu et al. 2012, 2013). Repeatedly it was isolated from solid-state fermentation materials used for the production of Maotai-flavor liquor (Song et al. 2019) but it was not found in all investigations on this kind of substrates (Wang et al. 2008; Xiu et al. 2012).

#### Other food and beverage related fermentations

*Schizosaccharomyces pombe* was found in the fermentation during production of Khadi, a traditional alcoholic beverage made from the fruit of *Grewia flava* (Malvaceae) in Botswana (Motlhanka et al. 2020). In traditional fermented foods in western Himalaya, *S. pombe* was detected (Pathania et al. 2010). *S. pombe* was isolated from agave molasses and fruit flies in a traditional tequila distillery (Lachance 1995) but not from mescal fermentation of *Agave salmiana* (Escalante‐Minakata et al. 2008) or the juice of *Agave tequilana* (Díaz-Montaño et al. 2008).

In conclusion *S. pombe* seems to be well adapted to fermenting environments. The manmade substrates in which it occurs regularly and as a main constituent of the yeast communities are characterized by some environmental conditions that mean a competitive advantage for *S. pombe* over the common strong fermenting yeast such as *Saccharomyces* spec.

### *Schizosaccharomyces octosporus*

#### Dried fruit

*Schizosaccharomyces octosporus* was described first by Beijerinck (1894) who obtained his culture from currants originating from Zante, Greece. In a later study Beijerinck (1897) repeatedly isolated *S. octosporus* in malt extract medium in which currants were incubated. Additionally, he found the yeast on figs from Smyrna but not on dates and raisins other than currants. Others reported the isolation of *S. octosporus* from sundried prunes (Phaff and Starmer 1980).

In this special context, the term “currants” requires some explanation. In English, currants can be both special raisins from Greece and the fruit of the genus *Ribes*. Beijerinck used German for the publications cited above. He isolated *S. octosporus* from “Korinthen” which are special small dark raisins from Greece. The fruit of *Ribes* spec. is called “Johannisbeeren” in German. “Korinthen” was later translated to “currants”. Thus, a reader who does not know the original text in German could be in doubt about which fruit Beijerinck actually investigated.

We detected *S. octosporus* in 88 of 430 dried fruit samples. It was isolated from 61 (42.1%) of 145 raisin samples and from 21 (40.4%) of 52 mango samples. From 18 samples of crabapple it was isolated 2 times. Of jackfruit and kaki only 1 and 3 samples were available on the market, respectively. From each fruit type *S. octosporus* was isolated once. Further investigations are needed in order to verify the frequency of *S. octosporus* in these fruit. Although a considerable number of samples of dried cherries (n = 16), apples (n = 10), apricots (n = 15), dates (n = 29), papaya (n = 13), plums (n = 21), pineapple (n = 26) and figs (n = 14) were investigated none or only 1 strain of *S. octosporus* was isolated from these fruit. The data show that *S. octosporus* is a typical member of the yeast communities on dried fruit but that its frequency varies considerably between different types of fruit. As in the case of *S. pombe* it remains to be investigated which factors favor the presence *S. octosporus* on some fruit and not on others.

The water activity (a_w_) in dried fruit is extremely low. It ranges between 60 and 68 (Tilbury 1980). As *S. octosporus* is highly osmotolerant it seems possible that it is able to proliferate on the fruit at least during drying when the water activity constantly degreases. Because it was isolated from fruit taken from closed plastic packages it is obviously able to survive on the fruit at least for several months.

With a frequency of 41.4% *S. octosporus* is most frequent on raisins among dried fruit in our study. Again this overall frequency could be misleading because of enormous differences between countries. In South African samples it was present in 20 (87%) of 23 samples. In raisin samples from Turkey and Greece which are neighboring countries 19 (43.2%) of 44 samples contained *S. octosporus* while in samples from Uzbekistan only 3 (11.1%) of 27 harboured the species. The reason for these marked differences remains unclear. For example, the way of drying, the kind of insects that visit the fruit in the vineyard or during drying or the local temperature regime could be factors that influence the frequency of *S. octosporus*.

As we know from our own experience fruit of all kinds sometimes mummify after full ripening on the plants and a lot of these fruit mummies remain on the plants until next spring. Mummified fruit could be seen similar to intentionally dried fruit although during mummification in nature decay proceeds. Sipiczki (2016) was able to show that several *Saccharomyces* species overwinter on mummified grapes in the Tokaya wine growing region in Hungary and Slovakia. Because *S. octosporus* in contrast to *Saccharomyces* species is hardly ever found on fresh fruit it seems questionable if the results of the above mentioned study can be transferred to *S. octosporus.*

#### Honey

Already Beijerinck (1897) tried to isolate *S. octosporus* from honey but he did not succeed. To our knowledge Lochhead and Farrell (1931) were the first who detected *S. octosporus* in honey from British Colombia, Canada. In their investigation only 1 of 191 Canadian honeys contained *S. octosporus*. Poncini and Wimmer (1986) isolated *S. octosporus* from 1 of 6 Fijian honeys. Also, it was isolated from honey in India (Kumbhojkar 1969, 1972) and Spain (Rodríguez-Andrade et al. 2019). From 2 honey samples out of 13 samples from northern China *S. octosporus* were isolated (Fan et al. 2014). *Schizosaccharomyces* species were detected in floral nectar and in the honey stomach of domesticated honey bees and wild bee species of the genera *Apis, Bombus, Andrena* and *Megachile* (Batra et al. 1973). Unfortunately, the authors did not determine their isolates to the species level. In contrast to these reports *S. octosporus* was not found in some other investigations in honey conducted in Portugal (Carvalho et al. 2005, 2010), Thailand (Saksinchai et al. 2012), Japan (Furuta and Okimoto 1978) or the USA (Fabian and Quinet 1928) indicating that *S. octosporus* is not omnipresent in honey like other species e.g. *Z. rouxii or Z. mellis*.

In the current study with a frequency of 16.3% *S. octosporus* was somewhat more frequent than *S. pombe* with a frequency of 12.7% in honey. As given above the data illustrated in Fig. 4 suggested that *S. pombe* and *S. octosporus* were more frequent in honeys coming from regions with longer vegetation periods according to the average yearly march of temperature (isothermomene). In the case of *S. octosporus* the differences in its frequency in honey assigned to different isothermomene categories were highly significant according to the Kruskal–Wallis H test (Kruskal and Wallis 1952). Therefore, it was tested if the occurrence of *S. octosporus* is somehow correlated with the different isothermomene categories. The Spearman-ρ correlation coefficient was low at 0.42 but highly significant (*p* < 0.01). This means that the occurrence of *S. octosporus* is more likely in a honey sample originating from regions with potentially longer vegetation periods than in those originating from cooler regions with potentially shorter vegetation periods. The reasons for this relation remains unclear but the results described in the literature point in a similar direction: While (Lochhead and Farrell 1931) found 1 strain of S. octosporus in their investigation including 191 honeys from Canada (Poncini and Wimmer 1986) isolated 1 strain from 6 honey samples from the Fijis.

Possibly, the frequency of yeasts generally is lower in cooler regions with potentially shorter vegetation periods or the special temperature requirements of *S. octosporus* are often not fulfilled in these regions. Further investigations on yeast density in honeys from different climatic regions and studies on the autecology of the single species are needed before justified conclusions can be drawn.

It was statistically tested if there is any correlation between the occurrence of one species and the other. For the occurrence of *S. pombe* and *S. octosporus* the spearman-ρ correlation coefficient was very low at − 0.17 but highly significant. This result suggests that occurrence of one species in a sample makes it more unlikely that the other species is present. This does not necessarily mean that both species directly interact. It cannot be totally excluded that the result is due to an experimental bias. In the case of honey, the enrichment medium was always streaked on 30% sugar medium. On this medium colony morphology is less pronounced. Colonies of many species just look smooth and glossy, exhibiting only minor differences. Additionally, in our experience some strains of both species can look similar under the microscope as long as no asci are present. Hence, one of the species could have been overlooked now and then if both species were actually present in an enrichment medium. This would lead to the effect that both species are a little rarer in those samples in which the other species occurs.

Also, some environmental factor that is in favor of one species and which is a disadvantage for the other could possibly have led to the negative correlation. Many factors may influence the presence and/or growth of the species eg the local temperature regime, the physical and chemical characteristics of the honey or just the way the beekeeper manages the hive.

Killer activities against *S. cerevisiae* and *C. glabrata* were described for some *S. pombe* strains (Bonilla‐Salinas et al. 1995). Thus, it cannot be excluded that *S. pombe* could suppress proliferation of *S. octosporus* to a certain degree.

Besides dried fruit honey was the only substrate from which *S. octosporus* was regularly isolated in the current study. As it is the case with *S. pombe S. octosporus* seems to be restricted to the honey and is usually not found in floral nectar. (Brysch-Herzberg 2004) could show that the yeast communities in bumblebee honey and floral nectar consist of highly specialized yeasts and that there is little overlap between both. In their study the authors did not find any *S. octosporus* strains in floral nectar. The isolation of obligate osmophilic strains in our study suggests that at least some strains of *S. octosporus* are restricted to habitats with an elevated osmotic pressure.

In the following discussion values for both the water activity and the moisture content are used because both terms are used in the literature. Earlier, several attempts were made to find a universal linear equation for a_w_ and MC (Chen 2019; Zamora et al. 2006) which all failed (Chen 2019) because a_w_ at a given MC changes significantly with many factors such as sugar composition (Gleiter et al. 2006), the state of crystallization (Zamora and Chirife 2006), or the type of honey (Gleiter et al. 2006).

Generally, honeys with a water content below 17.1% are regarded as safe from fermentation and those with water content of 20% and above are always in danger of fermentation, both irrespective of the yeast cell number per volume. In honeys with a moisture content between 17.1% and 20% the occurrence of fermentation depends on the cell number per volume (Mccleskey and Oertel 1950). Honeys with a_w_ values below 0.60 are regarded as to be microbiologically safe and those with a_w_ values above have a potential to undergo fermentation (Sanz et al. 1995).

After collection by honey bees floral nectar needs to lose about 80% of its moisture before it becomes honey with a water content of 17–20% (Sanz et al. 1995). Naturally, the length of the process depends on the nectar sugar concentration, the ambient humidity and the temperatures. In the case of floral nectar from *Vitex* spec. a duration of 10 days was reported (Wen et al. 2017), a period that seems long enough for a specialized highly osmotolerant yeast to effectively proliferate.

In earlier investigations it was shown that water content of 98 honeys from Morocco was between 13 and 24% (Terrab et al. 2002) and that of 44 Spanish honeys from Andalusia was between 15 and 19%. In 21 honeys from the north coast of Spain water contents between 15.8% and 22.2% were measured with an average of 18.7%. The authors state that the water content of two honeys only was in the range that means no risk for fermentation (Sanz et al. 1995).

Interestingly, it was shown that the moisture content in honey of single neighboring combs varies in the range of 2.5 percentage points (Mccleskey and Oertel 1950). The author reported areas of combs swollen due to fermentation in neighborhood of combs that are not effected by yeasts. The cell numbers in the swollen combs were in the range of 5 000 000 to 24,000,000 cells/ml whereas in non-fermenting combs the cell numbers were in the range of a few thousand up to 350,000 cells/ml. The results reported by Mccleskey and Oertel (1950) show that in order to judge if honey is a microhabitat for proliferation of certain yeasts it is not enough to just measure a_w_ or moisture content in the honey glass but that is necessary to explore single honey combs. A honey from the market may be assessed as microbiological stable because of a moisture content of 16.9%. Nevertheless, single combs in the hive may have fermented at higher moisture contents. This could be the reason why we isolated *S. octosporus* and other yeasts from numerous honeys although only one showed visible signs of fermentation.

Additionally, the moisture content of the liquid phase of honey rises when crystallization proceeds (Gleiter et al. 2006). Experimental data show that during crystallization a_w_ changes of about 0.03 to 0.04 occur. Thus a honey which is initially microbiological stable due to an a_w_ of 0.58 may start fermentation after crystallization at an a_w_ of 0.62 (Shafiq et al. 2012; Zamora and Chirife 2006).

The range and dynamics of honey water content reported in the literature cited above make clear that honey combs in bee hives are habitats in which *S. octosporus* at least temporarily may propagate. Either the water content in the single honey combs is permanently above the level that allows *S. octosporus* to grow or it is above this level for the time of honey ripening which could leave *S. octosporus* enough time for proliferation. Additionally, factors like crystallization, ambient humidity or the temperature cause relevant changes in the a_w_ of honey and thus lead to temporarily favorable conditions for *S. octosporus*. Because of its high osmotolerance (Vaughan-Martini and Martini 2011). *S. octosporus* seems to be better suited for proliferation in the low a_w_ environment in honey than *S. pombe* which could be the reason for the somewhat higher frequency of *S. octosporus*.

The authors have observed masses of honey bees in September on overripe Müller-Thurgau grapes in Germany. It is well known that honey bees utilize fruit juices and plant exudates in the case floral nectar is rare (Standifer 1980). Therefore, it seems possible that *S. octosporus* as well as *S. pome* is carried to ephemeral habitats such as drying or dry fruit by the honey bees from time to time.

#### Solitary bee beebread

*S*train CBS 7191 is the only *S. octosporus* strain that we are aware of that was isolated from the provisions of solitary bees (here the mason bee *Osmia rufa*). In the current study from 123 samples of solitary bee beebread of different solitary bee species *S. octosporus* was isolated once. We conclude that solitary bee beebread is most likely of little importance as natural habitat of *S. octosporus*.

#### Other substrates

In the current study *S. octosporus* was not detected in 61 grape samples collected in the vineyard, 22 samples of various fruit and 11 samples of composted apples. All these samples were incubated in medium FG that should give *S. octosporus* a selective advantage about many other yeasts species. Thus, it seems justified to presume that fresh fruit are of little importance for *S. octosporus* as habitat.

### *Schizosaccharomyces osmophilus*

#### Solitary bee beebread

Solitary bees’ provisions consist of a mixture of pollen and floral nectar. The proportions of both constituents vary in a wide range depending on the bee species and the food source that is available (Danforth et al. 2019). Batra et al. (1973) isolated some *Schizosaccharomyces* strains which were not further determined, from the provisions of solitary bees. Principally, bee bread should support the growth of osmotolerant yeasts because nectar and pollen offer a rich mixture of simple sugars, proteins and minerals. Solitary bee beebread was investigated from two locations which are in a distance of about 150 km from each other in southwestern Germany. *S. osmophilus* was isolated at both locations in June and July only although starting from April solitary bee beebread was investigated regularly. From 64 samples taken from end of March until end of May in three consecutive years only 1 strain (1.6%) of *S. osmophilus* was isolated. The sample containing *S. osmophilus* was collected at mid-May. Of 49 samples collected in June and July in the same years 13 (26.5%) harbored *S. osmophilus*. This analysis includes the results reported by Brysch-Herzberg et al. (2019). The repeated isolation of the species from beebread at different locations in different years suggest that solitary bee beebread is a typical habitat of *S. osmophilus*. The fact that it was only detected in the substrate in summer and not in early spring provokes the question where the yeast lives in the year round. Further investigations are needed to answer this question.

#### Dried fruit and honey

From 386 honey samples *S. osmophilus* was isolated once and from 154 raisin samples two strains were isolated. The honey sample from which it was isolated came from Spain and the raisin samples came from Uzbekistan and Iran. From 18 samples of crabapple originating from China *S. osmophilus* was isolated two times. Brysch-Herzberg et al. (2019) report isolation of *S. osmophilus* from a Spanish fig snack. Therefore, it can be concluded that *S. osmophilus* is distributed over the whole Eurasian Continent from Spain to China. Obviously its osmophilic nature restricts it to substrates that have an elevated osmotic pressure. Further investigations are needed in order to understand its life history in the course of the year.

### *Schizosaccharomyces japonicus*

#### Fresh fruit

Coker and Wilson (1911) isolated a yeast strain from Delaware grapes bought in Chapel Hill, USA. They were of the opinion that the yeast was *S. octosporus*. The drawings they made show vegetative cells and asci rather typical for *S. japonicus* which was not described at that time. On grapes of *Vitis davidii*, *S. japonicus* was shown to be one of four dominating yeast species in Guizhou, China (Wang et al. 2019). *S. versatilis* was described by Wickerham and Duprat (1945), #340 on the basis of strains which were isolated from home-canned grape juice that started fermentation. Currently, *S. versatilis* is seen as a synonymous to *S. japonicus. S. japonicus* strain CBS 7116 was isolated from grape must by C. Delfini in Lombardia, Italy and *S. japonicus* strains CLIB 831, CLIB 838 and CLIB 847 were isolated from substrates related to wine making. Fittingly, CBS 2629 originates from a Portuguese white wine. Additionally, the yeast was detected in all stages of spontaneous fermentations of grape juice in a study based on next generation sequencing (NGS) in Portugal (Pinto et al. 2015). In other studies using NGS approaches *S. japonicus* was not found on grapes (Morgan et al. 2017). In the current study *S. japonicus* was not isolated from grapes but from a mummified plum, from the bark of fruit-bearing trees outside the forest and from soil from under an apple tree. The type strain of *S. japonicus* (NBRC 1609) was derived from fermenting berries. We conclude that *S. japonicus* occurs regularly but infrequently on fruit.

#### Forest materials

In 1927, near St. Petersburg, Krassilnikow isolated *S. japonicus* from slime flux of *Quercus* spec (Kudrjawzew 1960). In Japan, Yoneyama (1957) isolated *S. japonicus* from sap exudates of *Pinus* spec., soil under *Pinus* spec. and bark of *Quercus variabilis*. Additionally, *S. japonicus* was isolated from numerous deciduous trees in Japan (Kodama 1974). Phaff et al. (1964) found *S. japonicus* in flux material of *Ulmus carpinifolia* in Davis, California yet described it as a species that was just periodically present in their 9-month investigation. During an investigation on yeasts associated with *Drosophila* species in an oak-pine forest in Ontario, Canada, *S. japonicus* was isolated (Lachance et al. 1995). It is well known that some *Drosophila* species feed on slime fluxes of trees (Phaff et al. 1964; Shehata et al. 1955). In the current study, *S. japonicus* was not isolated from forest samples. However, *S. japonicus* was isolated in our earlier isolation trials performed with medium O in the sampling areas Ruwer-Valley and Oberolmer Forest (see Brysch-Herzberg and Seidel 2017), Germany and in Provence, France. Different forest materials like bark, sap flux or soil harbor *S. japonicus*. In agreement with the literature cited above, we conclude that *S. japonicus* is a typical member of forest yeast communities although it is not as frequent as *Sac. paradoxus* and other yeasts. The isolation of *S. japonicus* from forest material in Provence which is characterized by heat and drought in summer suggests that *S. japonicus* can not only be isolated from the temperate and humid forest of Germany but from forests in other climate zones, too.

#### Other substrates

A few reports exist about the occurrence of *S. japonicus* on substrates other than fruit and forest materials. Fahrasmane et al. (1988) isolated *S. japonicus* from a spontaneous sugar cane sap fermentation in a Haitian rum distillery. Strain MUCL 55012 was isolated from “chicha de jora” which is a traditional corn beer from Peru. Thus, *S. japonicus* seems to have a much broader ecological plasticity compared to *S. octosporus* or *S. osmophilus*. Like *S. pombe S. japonicus* seems to be well adapted to strong fermenting environments. Fittingly, we have experienced during our own oenological trials that some strains of *S. japonicus* almost reaches the fermentation capacity of *S. pombe* (data not shown).

## General conclusions

In the current study, 226 strains of *S. pombe*, 150 strains of *S. octosporus*, 9 strains of *S. osmophilus* and 22 strains of *S. japonicus* were isolated. In the following, the main findings are summarized.

### *Schizosaccharomyces pombe*

*Schizosaccharomyces pombe* is regularly but infrequently present on some ripe fresh fruit like grapes and apples. Additionally, it can be found on dried fruit such as mangos, pineapple and raisins among others. The only natural substrate in which the species was detected regularly was honey bee honey. The significant variability of moisture content and water activity in single honey combs, in honey produced in different seasons and different regions as well as the changes in moisture and water activity over the time of ripening make honey a natural substrate in which the species potentially could propagate at least temporarily. The presence of *S. pombe* on fruit could be explained by carryover of some *S. pombe* cells from the hive to the fruit by foraging honey bees. With its high tolerance against fermentation products like acetic acid or ethanol its seems to be well adapted to substrates heavily influenced by fermentation which regularly occurs in honey combs.

### *Schizosaccharomyces octosporus*

*Schizosaccharomyces octosporus* is a species well adapted to low a_w_ environments like honey and dried fruit. It is rarely found in any other substrates. Its outstanding high osmotolerance suggests that it is able to propagate in honey at least locally in some single high a_w_ combs and temporarily in seasons with generally higher a_w_ of honey or during honey ripening when a_w_ is lower and slowly rises. To strong fermenting environments *S. octosporus* seems to be poorer adapted than *S. pombe* because of its low tolerance to acetic acid and lower fermentation power. Nonetheless, its ability to start fermentation and propagation in a medium containing 8% ethanol place *S. octosporus* among the yeast species with a high ethanol tolerance. In connection with honey as a principal habitat which frequently starts fermentation in the combs this seems to be an advantageous adaptation.

### *Schizosaccharomyces osmophilus*

In very rare cases *S. osmophilus* was isolated from honey bee honey and from raisins. Solitary bee beebread was the substrate from which *S. osmophilus* was isolated with the highest frequency. Naturally, its obligate osmophily makes *S. osmophilus* a species that depends on substrates with a constantly reduced a_w_. Further research is required to judge if *S. osmophilus* somehow lives together with solitary bees in the turn of the year.

### *Schizosaccharomyces japonicus*

Like *S. pombe S. japonicus* has a high fermentation power and ethanol tolerance. On the other hand, *S. japonicus* is the only *Schizosaccharomyces* species that is regularly isolated from different forest materials. It is the only *Schizosaccharomycs* species with a rather low osmotolerance and has the ability to grow with true hyphe which is a unique feature in the genus. These characteristics suggest that *S. japonicus* possesses a fundamentally different life history than the other species in the genus *Schizosaccharomyces*.

### *Schizosaccharomyces cryophilus*

*Schizosaccharomyces cryophilus* is the only *Schizosaccharomyces* species that was not isolated in the current study. Only one strain of the species is known by now which co-existed with *S. octosporus* in the material of strain CBS 7191. This strain was isolated from solitary bee (*Osmia rufa*) bee bread (Helston et al. 2010). As the true origin of the strain is obscured no conclusions can be drawn from these facts about its ecology.

## Supplementary Information

Below is the link to the electronic supplementary material.

Supplementary file1 (XLSX 12 kb)

Supplementary file2 (XLSX 16 kb)

Supplementary file3 (XLSX 11 kb)

## Data Availability

All relevant data are provided in the manuscript, tables, figures and supplementary tables.
